# Overactivated sonic hedgehog signaling aggravates intrauterine adhesion via inhibiting autophagy in endometrial stromal cells

**DOI:** 10.1038/s41419-020-02956-2

**Published:** 2020-09-15

**Authors:** Cheng Wei, Yibin Pan, Yinli Zhang, Yongdong Dai, Lingling Jiang, Libing Shi, Weijie Yang, Shiqian Xu, Yingyi Zhang, Wenzhi Xu, Yanling Zhang, Xiaona Lin, Songying Zhang

**Affiliations:** 1 Assisted Reproduction Unit, Department of Obstetrics and Gynecology, Sir Run Run Shaw Hospital, School of Medicine, Zhejiang University, Hangzhou, 310016 China; 2 Key Laboratory of Reproductive Dysfunction Management of Zhejiang Province, No. 3 Qingchun East Road, Jianggan District, Hangzhou, 310016 China

**Keywords:** Autophagy, Reproductive disorders, Pathogenesis

## Abstract

Autophagy can be dynamically induced in response to stresses and is an essential, ubiquitous intracellular recycling system that impacts the fate of damaged resident cells, thereby influencing wound healing. Endometrial fibrosis is a form of abnormal wound healing that causes intrauterine adhesion (IUA) and infertility. We previously demonstrated that overactivated sonic hedgehog (SHH) signaling exacerbated endometrial fibrosis, but the role of autophagy in this process is still unknown. Here, we report that impaired autophagy participates in SHH pathway-induced endometrial fibrosis. Endometrial stroma-myofibroblast transition accompanied by autophagy dysfunction was present in both endometrial biopsies of IUA patients and *Amhr2*^*cre/+*^ *R26-SmoM2*^*+/−*^ (AM2) transgenic *mouse*. Mechanistically, SHH pathway negatively regulated autophagy through pAKT-mTORC1 in a *human* endometrial stromal cell line (T-HESCs). Furthermore, SHH pathway-mediated fibrosis was partly counteracted by autophagy modulation in both T-HESCs and the *murine* IUA model. Specifically, the impact of SHH pathway inhibition (GANT61) was reversed by the pharmacological autophagy inhibitor chloroquine (CQ) or RNA interference of autophagy-related gene *ATG5* or *ATG7*. Similar results were obtained from the *murine* IUA model treated with GANT61 and CQ. Moreover, promoting autophagy with rapamycin reduced fibrosis in the AM2 IUA model to baseline levels. In summary, defective autophagy is involved in SHH pathway-driven endometrial fibrosis, suggesting a potential novel molecular target for IUA treatment.

## Introduction

The human endometrium is a highly dynamic tissue that serves as a “fertile ground” for embryo implantation and placenta development^1^. Because the endometrium is a fertility-determining factor^2^, intrauterine adhesion (IUA), which has increasing incidence due to increasing trauma to the gravid uterus, is the second leading cause of female secondary infertility.

IUA mainly manifests as endometrial fibrosis and is considered an abnormal wound healing process after damages beyond the tissue’s scar-less regenerative ability, regardless of traumatic injury or infectious damage^3^. IUA is characterized by excessive avascular fibrous tissue, spindle-shaped myofibroblasts, and inactive cubocolumnar glands in the place of normal stroma and epithelium^4^. Notably, excessive extracellular matrix (ECM) deposition is not just the outcome of endometrial fibrosis but can also exacerbate fibrosis through its stiffness^5,6^. Presently, myofibroblasts are the major ECM-producing cell^6^, and genetic cell fate tracing studies have revealed that resident stromal cells are the predominant origin of myofibroblasts in multiple organic fibrosis^7–9^.

Autophagy is a highly conserved degradation and recycling system within cells that is gentle under physiological conditions and reacts adaptively to various stimuli^10,11^. Autophagy initiates the formation of autophagosomes sequestering unnecessary elements, followed by fusion with lysosomes to form autolysosomes and degradation by lysosomal hydrolases^12^. This process saves energy and removes toxicant, thereby facilitating cellular adaptation to stress^12^, however, whether it is beneficial or harmful for an organism is strongly context-dependent^13^. Mounting evidence indicates that dysfunctional autophagy is closely associated with excessive ECM deposition and fibrogenesis. Studies have reported that disrupting autophagy-related genes (*ATG5, ATG4B,* and *MAP1S*) leads to fibrotic protein accumulation in the kidney^14,15^ and lung^16^, whereas the role of autophagy in endometrial fibrosis is still unclear.

The hedgehog (HH) pathway is a conserved morphogenic pathway that is essential for all life stages, from embryonic development to adult tissue maintenance^17^. In humans, sonic hedgehog (SHH), one of the most widespread HH ligands and a long-range morphogen^18^, can be secreted and bind to the 12-transmembrane glycoprotein receptor patched 1 (PTCH1), inducing activation of the 7-transmembrane G protein-coupled protein smoothened (SMO)^19^. These events facilitate its downstream effectors, GLI family zinc finger (GLI) transcription factors, which enter the nucleus and regulate transcription of corresponding genes, such as *PTCH1* and *GLI1*^20^, whose expression also reflects HH pathway status. Numerous studies have demonstrated that the HH pathway induces fibrosis in many organs^7,21–27^. We previously found that expression of the HH effector *GLI2* was significantly increased in the endometrial stroma of IUA samples and was related to fibrosis severity^28^, indicating aberrant activation of the HH pathway in connection with endometrial fibrosis. However, in vitro results indicated that the HH inhibitor reduced fibrotic protein expression, collagen I, and connective tissue growth factor (CTGF), in human endometrial stromal cells at 48 h (>25%)^28^, while these changes were not fully attributable to the corresponding mRNA expression changes (<25%).

Increasing evidence has demonstrated that HH signaling regulates autophagy in *drosophila*, *mammalian* healthy cells or cancer cells, despite controversial findings^29–34^. Thus, we hypothesize that SHH pathway can regulate protein degradation systems (specifically autophagic degradation for stress adaptation) to utilize energy and materials more efficiently during wound healing. Thus, we explored whether autophagy influences endometrial fibrosis, how SHH pathway regulates autophagy in endometrial stromal cells and whether autophagy is involved in SHH-induced-endometrial fibrosis.

## Results

### Autophagy levels vary in endometrial stromal cells in different endometrial fibrosis locations

We collected endometrial samples from normal (*n* = 5) and IUA (*n* = 9) donors and assessed autophagy in the stroma by location, analyzing the normal endometrium and fibrous lesions, boundary endometrial tissue, and detached endometrial tissue in IUA samples.

**Fig. 1:**
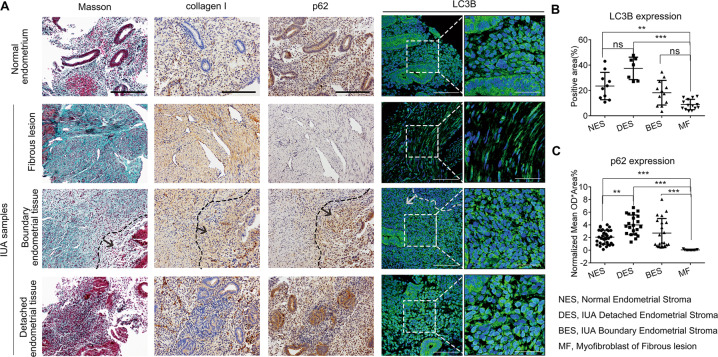
Autophagy levels varied in different regional endometrial stroma among endometrial fibrosis. **a** Representative images of Masson, collagen I, and p62 IHC staining and LC3B immunofluorescence staining in normal endometrium and different locations in IUA samples (including fibrous lesions, boundary endometrial tissue, and detached endometrial tissue). Scale bars, 200 μm (1st–3rd), 50 μm (4th), 20 μm (5th), and 1 μm (6th column). Dotted lines indicate the boundary of the fibrous lesion, and arrowheads point to boundary endometrial tissue. **b**, **c** Quantitative analyses of the %area of LC3B puncta (**b**) and mean ODx the % positive area of p62 (**c**) in different endometrial stroma. Each scatter plot represents data from a high magnification field. All data were obtained from five normal endometrial patients and nine IUA patients. The data represent the mean ± S.D. NES normal endometrial stroma, BES IUA boundary endometrial stroma, DES IUA-detached endometrial stroma, MF myofibroblast of fibrous lesion. Statistical analyses were performed by Kruskal–Wallis test (nonparametric method) plus Dunn’s multiple comparisons test (**b**, **c**). ***p < 0.001; **p < 0.01; *p < 0.05; ns not significant.

Autophagy markers were significantly lower in myofibroblast in the fibrous lesion, than that in normal endometrial stroma, as indicated by LC3B fluorescence (8.88 ± 4.09% vs. 23.47 ± 10.79%, *p* < 0.001) and p62/SQSTM1 coloration (0.04 ± 0.06 vs. 2.06 ± 1.11, *p* < 0.001). This autophagic dysfunction colocalized with marked ECM accumulation, as indicated by Masson and collagen I immunohistochemical (IHC) staining (Fig. 1a). Consistently, in three different sites of IUA samples, autophagy declined gradually from detached endometrial stroma, to boundary endometrial stroma, then myofibroblast in the fibrous lesion, as indicated by LC3B (37.42 ± 8.86%, 18.19 ± 9.73%, and 8.88 ± 4.09%, respectively) and p62 (4.00 ± 1.56, 2.70 ± 2.30, and 0.04 ± 0.06, respectively) expression. Additionally, autophagy indices were higher in detached endometrial stroma than in normal endometrial stroma. All LC3B and p62 expression levels above were quantitatively analyzed by ImageJ and are displayed as scatter plots (Fig. 1b, c).

These results indicate that autophagy levels varied in distinct regions in the endometrial stroma of IUA samples and were related to ECM expression, indicating the essential roles of autophagy in endometrial regeneration and fibrosis progression.

### SHH signaling negatively regulates autophagy initiation

**Fig. 2:**
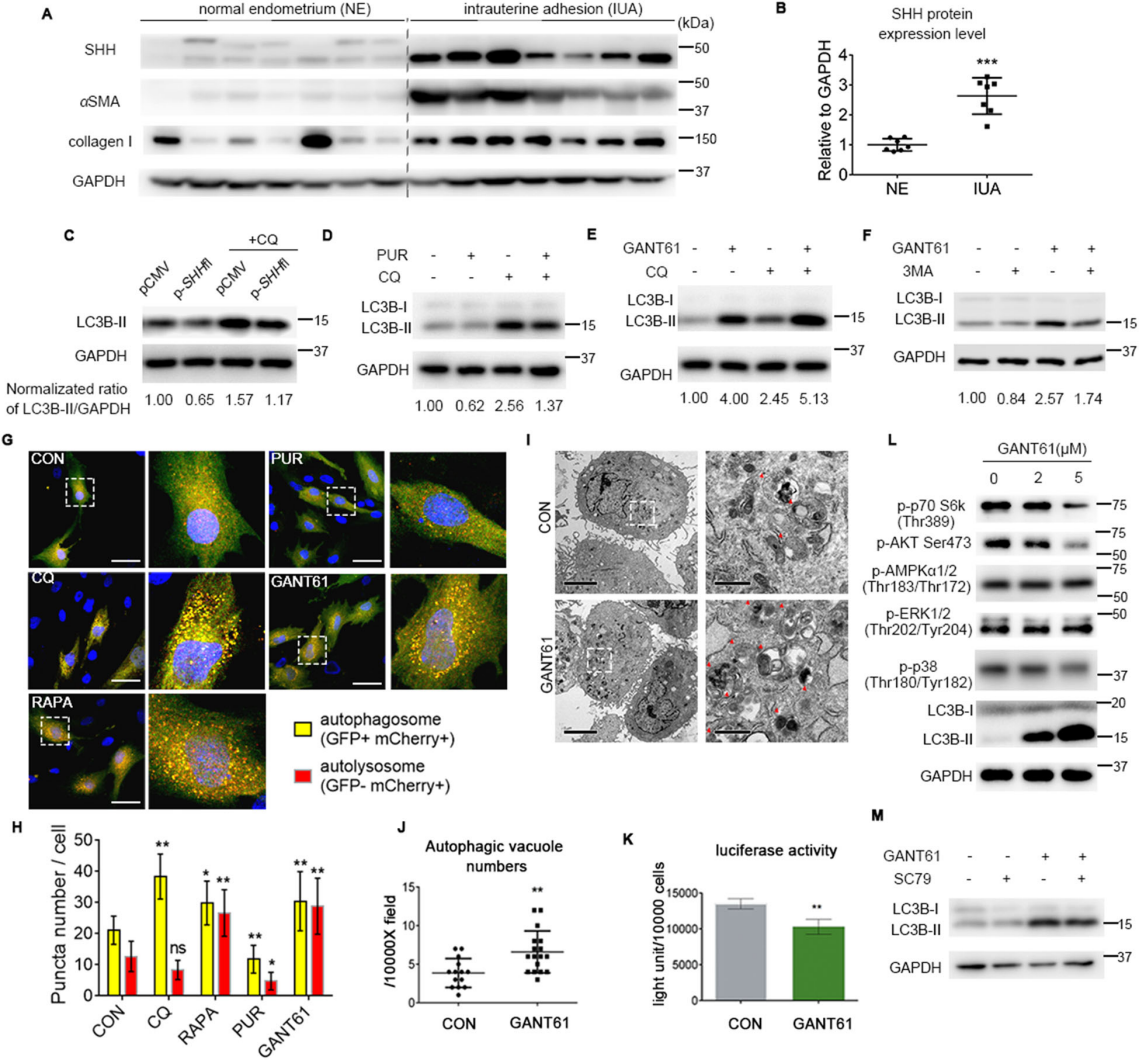
SHH signaling negatively regulates autophagy initiation. **a** Representative immunoblot images of SHH (full length) and fibrotic markers (*α*SMA and collagen I) in normal endometrium (NE) and intrauterine adhesion (IUA) sample lysates. **b** Semiquantitative plot showing SHH expression relative to GAPDH and normalized to the normal endometrium group. **c** T-HESCs were transfected with pCMV (vector) or p*SHH*fl plasmid and then treated with CQ (20 μM) for the last 4 h before being collected for western blot analysis. **d**, **e** T-HESCs were treated with the SMO agonist purmorphamine (**d**, PUR) or the GLI inhibitor (**e**, GANT61) for 24 h, followed by treatment with or without CQ (20 μM) for 4 h before being collected for western blot analysis. **f** T-HESCs were treated with GANT61 with or without 5 mM 3-MA for 6 h. LC3B-II levels were quantified by densitometric analysis, were normalized to GAPDH, and are shown under the indicated bands. **g** After being infected with Ad-mCherry-GFP-LC3B, T-HESCs were treated with SHH drugs (PUR 5 μM and GANT61 5 μM) for 24 h, the autophagy drug CQ 20 μM for 4 h or RAPA 0.1 μM for 2 h before confocal fluorescence analysis. Scale bars, 50 µm. **h** Relative quantifications of yellow vesicles (GFP^+^mCherry^+^ puncta and autophagosomes) and red vesicles (GFP^−^mCherry^+^ puncta and autolysosomes) per cell by ImageJ software; cells were treated as described in **g**. Pictures were taken from three independent experiments. Each group had a cell number >15. The data represent the mean ± S.D. **i** TEM images of T-HESCs with or without GANT61 treatment for 24 h. Right-side images are relative enlargements of the dotted box on the left. Red triangles indicate autophagic vacuoles. Scale bars, 5 μm and 1 μm (left and right, respectively). **j** Quantification of autophagic vacuoles per 10,000X field of three independent experiments; cells were treated as described in **h**. The data represent the mean ± S.D. **k** T-HESCs were transfected with the p-Luc2p-p62 vector, and 24 h later, the cells were treated with vehicle or GANT61 (5 µM) for 24 h before cell extracts were prepared for firefly luciferase activity. The light unit was normalized by relative cell number (10000 cells). Data represent the mean ± S.D. of three independent experiments. **l** Western blot analysis of p-p70 S6k (a substrate of mTORC1) and known mTOR upstream modulators (p-AKT Ser473, p-AMPK*α*, p-ERK1/2, and p-p38) in protein lysates of T-HESCs after GANT61 treatment for 24 h. **m** T-HESCs were treated with GANT61 (5 µM) with or without SC79 (10 μM) for 24 h before western blot analysis of LC3B expression. Statistical analyses were performed by two-sided Mann–Whitney *U*-test (**b**, **j**) and two-way ANOVA plus Bonferroni’s multiple comparisons test (**h**) comparing to group control. **p < 0.01; *p < 0.05; ns not significant.

An accepted model of HH-regulated fibrosis is that after injury, excessive HH ligand is released^22,24^ and acts on nearby target cells. The expression of full-length SHH ligand was much higher in IUA samples (*n* = 7) than in normal endometrium (*n* = 7) (Fig. 2a, b), together with elevated expression of ECM proteins *α*SMA and collagen I. Additionally, the PTCH1 receptor was expressed in the cytoplasm and nucleus of endometrial stromal cells (Supplementary Fig. 1A). Because the transcriptional activator GLI2 is elevated in the stroma of IUA tissues^28^, and autophagy dysfunction was evident in the study described above, we further explored the relationship between SHH signaling and autophagy in a *human* endometrial stromal cell line, T-HESCs.

**Fig. 3:**
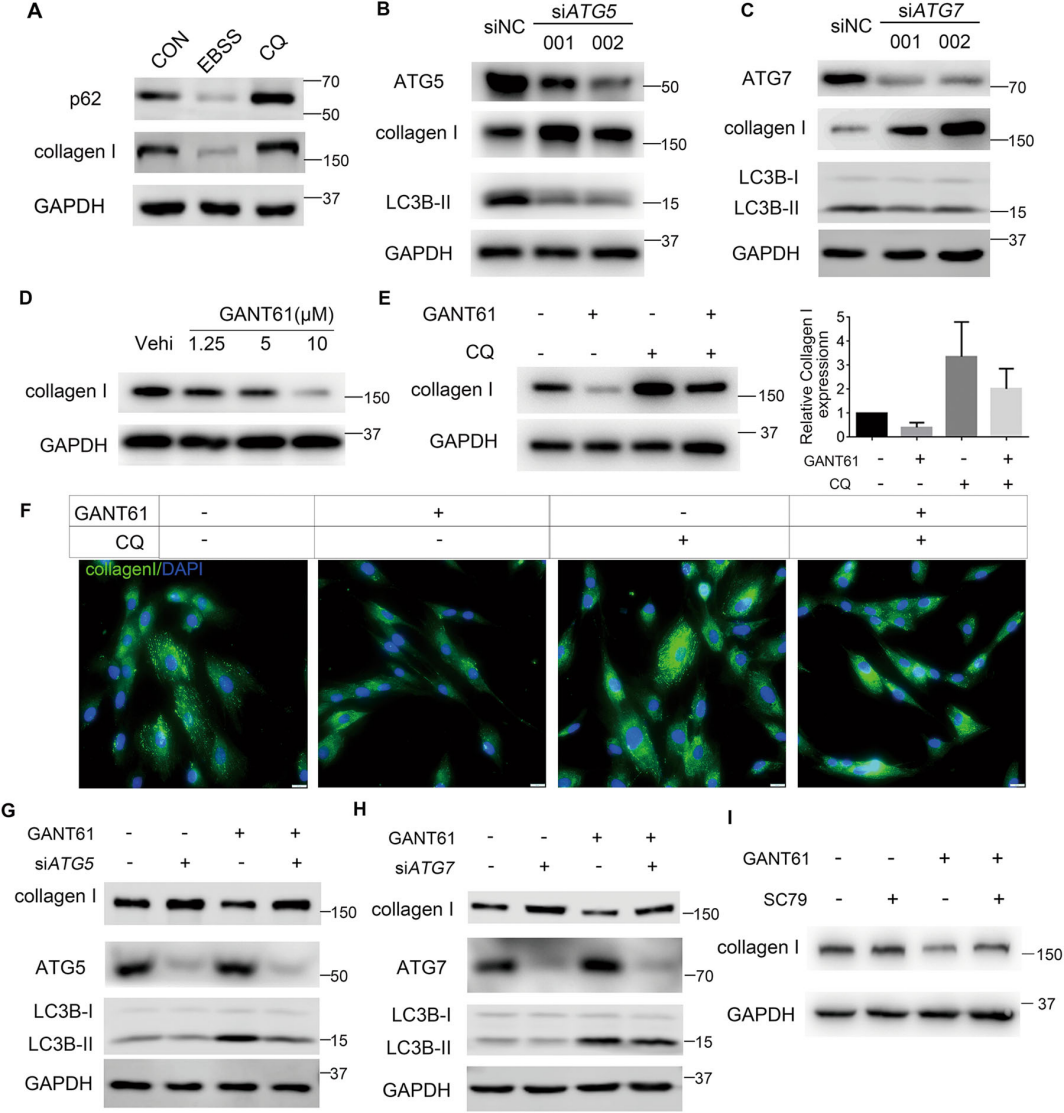
Autophagy reversed the effect of the SHH pathway on collagen I in T-HESCs. **a** Representative immunoblot images of p62 and collagen I in T-HESCs treated with an autophagy stimulator (EBSS) for 4 h or a lysosomal inhibitor (CQ 20 μM) for 8 h. **b**, **c** T-HESCs were transfected with control (siNC), siATG5 (**b**), or siATG7 (**c**) for 48 h before being collected for immunoblot analysis to measure collagen I, LC3B, and ATG5 (or ATG7) expression. **d** Representative immunoblot images of collagen I in T-HESCs treated with gradient doses of GANT61 for 24 h. **e** T-HESCs were treated with GANT61 for 24 h, followed by treatment with or without 20 μM CQ for 4 h before being collected for immunoblot analysis. The right bar chart is normalized relative expression of collagen I from three independent experiments, as indicated on the left side. The data represent the mean ± S.D. **f** Representative images showing immunostaining of collagen I (green) in T-HESCs treated as described in **c**. Nuclei are labeled with DAPI and are blue. Scale bar, 100 µm. **g**, **h** T-HESCs were transfected with control (siNC), siATG5 (**e**) or siATG7 (**g**) for 48 h before being collected for immunoblot analysis of collagen I, LC3B, ATG5, or ATG7. **i**, T-HESCs were treated with GANT61 (5 µM) with or without SC79 (10 µM) for 24 h before western blot analysis of collagen I expression.

Transfection of p*SHH-*fl decreased autophagy marker LC3B-II expression compared with its vector plasmid (Fig. 3a). The SMO agonist purmorphamine (PUR) reduced LC3B-II expression, while the GLI inhibitor GANT61 increased LC3B-II expression in a graded manner (Supplementary Fig. 1C, D). The effects of p*SHH*-fl transfection or PUR or GANT61 treatment on HH target gene mRNA (*GLI1* and *PTCH1*) are also shown (Supplementary Fig. 1B, E).

Autophagic flux is a dynamic process from initiation to degradation, and LC3B-II accumulation could be caused by autophagosome formation or degradation blockade. Measuring LC3B-II turnover in the absence or presence of chloroquine (CQ), which increases lysosomal pH and blocks autophagic degradation, can distinguish the above two causes^35^. Relative LC3B-II expression was measured after treatment with drug or CQ and indicated that PUR decreased autophagic flux (Fig. 2d), while GANT61 increased autophagic flux (Fig. 2e) compared to vehicle. Furthermore, the PI3K inhibitor 3-methyladenine (3-MA), which inhibits autophagy initiation^36^, mostly reversed GANT61-induced LC3B-II accumulation (Fig. 2f). Ad-mCherry-GFP-LC3B was used to differentiate autolysosomes (GFP^−^ mCherry^+^ vesicles) from autophagosomes (GFP^+^ mCherry^+^ vesicles) due to differing instabilities of GFP and mCherry fluorescent proteins in autolysosomes^37^. Consistent with the LC3B-II turnover results, autophagosome and autolysosome numbers increased after GANT61 treatment but decreased after PUR treatment relative to those of the control (Fig. 2g, h). Furthermore, there were more autophagic vacuoles after GANT61 treatment compared with the vehicle control, as determined by transmission electron microscopy (TEM; Fig. 2i, j). These data indicate that SHH signaling impacted autophagy initiation.

Additionally, GANT61 decreased the luciferase activity in lysates from T-HESCs transfected with the Luc2p-p62 plasmid, which expresses a recombinant p62 protein fused with a luciferase variant of Luc2p at the N-terminus to distinguish it from endogenous p62^38^, suggesting that GANT61 accelerated Luc2p-p62 degradation (Fig. 2k).

In summary, in T-HESCs, SHH pathway negatively modulated autophagy initiation.

### AKT-mTOR signaling contributes to SHH-mediated inhibition of autophagy initiation

We next explored whether SHH signaling regulated autophagy initiation via mechanistic target of rapamycin complex I (mTORC1), a classic suppressor of autophagy initiation, and measured the phosphorylation level of p70 ribosomal S6 protein kinase (p70S6K), a substrate of mTORC1.

GANT61 treatment caused a significant graded decline on p-p70S6K level (Fig. 2l), indicating decreased mTORC1 activity. Further detection of mTOR upstream modulators revealed that Ser473 phosphorylation levels on AKT decreased notably after GANT61 treatment (Fig. 2l), while others (p-AMPK*α*, p-ERK1/2, and p-p38) did not obviously change (Fig. 2l) in a graded manner similar to those of LC3B-II. Moreover, the AKT activator SC79 partially counteracted the GANT61-induced change in LC3B-II levels (Fig. 2m). SC79-mediated activation of p-AKT Ser473 was confirmed by immunoblotting analysis of p-AKT Ser473 and its substrate p-GSK3*β* Ser9 (Supplementary Fig. 1F). These data show that the SHH pathway partially regulated autophagy initiation through the pAKT-mTORC1 axis.

### Autophagy reverses the effect of the SHH pathway on collagen I

We evaluated whether autophagy participated in SHH pathway-mediated endometrial fibrosis via autophagic degradation of fibrotic markers. Due to the intricate reciprocal relationship between TGF*β*1 signaling and autophagy^39,40^, we performed experiments in a pure T-HESCs system without TGF*β*1 treatment.

We found that the crucial fibrotic marker collagen I decreased after treatment with the autophagy stimulator Earle’s salts for 4 h but increased after treatment with the autophagy inhibitor CQ for 8 h, similar to the effects on p62 (Fig. 3a). Other fibrotic proteins (*α*SMA, CTGF, fibronectin 1, vimentin, and collagen VI) did not undergo the same pattern of changes (Supplementary Fig. 3a) in this context of transient autophagic modulation. In addition, collagen I accumulated when autophagy-related genes, including autophagy-related 5 *(ATG5*; Fig. 3b) or autophagy-related 7 (*ATG7*; Fig. 3c), were knocked down in T-HESCs, together with the decline in LC3B-II. These results indicate that collagen I was sensitive to autophagy regulation in the T-HESC system.

Moreover, changes in collagen I and LC3B-II after SHH signaling modulation were negatively correlated; GANT61 treatment reduced collagen I and induced LC3B-II expression (Fig. 3d), and PUR had the opposite effects (Supplementary Fig. 3B).

Furthermore, CQ reversed the GANT61-mediated reduction in collagen I expression, as shown by immunoblotting and immunofluorescence (Fig. 3e, f). A parallel experiment indicated that the mRNA levels of *COL1A1* and *COL1A2*, two genes encoding collagen I chains, changed slightly (Supplementary Fig. 3C). Similar reversion of GANT61-reduced collagen I expression were achieved by knockdown of *ATG5* (Fig. 3g) or *ATG7* (Fig. 3h) or with SC79 treatment (Fig. 3i).

Collectively, autophagy modulated SHH-endometrial fibrosis in part by mediating fibrotic protein degradation.

### Autophagy reverses the effects of the SHH pathway on *murine* endometrial fibrosis

**Fig. 4:**
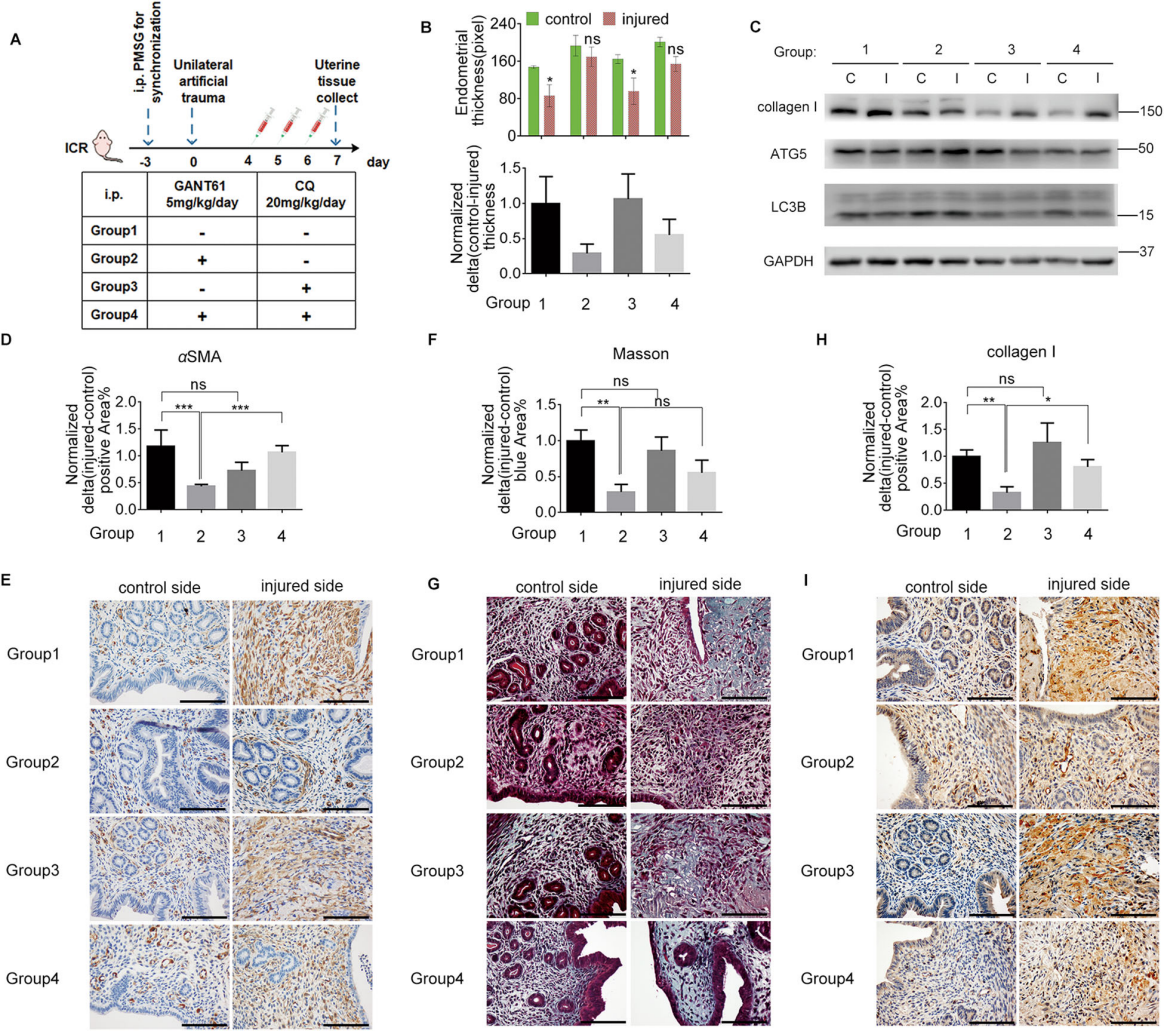
Autophagy reversed the effects of the SHH pathway on murine endometrial fibrosis. **a** Schematic diagram of the groupings and detailed treatments with GANT61 and CQ in a *murine* IUA model. **b** The top panel shows the thickness of the normal endometrium in both the control and injured side in the four groups (5 mice/group); statistical analyses were performed comparing the control and injured sides and are shown in this graph. The bottom panel shows the corresponding normalized changes in endometrial thickness in the top panel. **c** A section of uterine lysate from groups 1–4 were analyzed by immunoblotting to measure collagen I, ATG5, LC3B, and GAPDH expression levels. **d**, **f**, **h** Quantitative analyses of the percentage of the *α*SMA-positive area (**d**), blue area showing Masson staining (**f**) and the collagen I-positive area (**h**) via normalized changes in each group. **e**, **g**, **i** Representative images of *α*SMA IHC staining (**e**), Masson staining (**g**), and collagen I IHC staining (**i**) in injured and control transverse uterine sections from mice in groups 1–4. Scale bars, 100 μm. Data show mean ± S.E. Statistical analyses were performed by two-way ANOVA plus Bonferroni’s multiple comparisons test (based on matching design) (**b**) and Kruskal–Wallis test (nonparametric method) plus Dunn’s multiple comparisons test (**d**–**f**) comparing to group control. ***p < 0.001; **p < 0.01; *p < 0.05; ns not significant.

For in vivo experiments, we generated a unilateral *murine* IUA model, followed by treatment with GANT61 or CQ. An experimental schematic diagram is presented in Fig. 4a. Herein, we defined differences between the injured and control uterine sides of the same *mouse* as changing values, which were referred to as comparative indices between different groups.

GANT61 treatment reduced the changes in endometrial thickness (Fig. 4b), *α*SMA expression (Fig. 4d, e), Masson staining (Fig. 4f, g), and collagen I expression (Fig. 4c, h, i) relative to those in the control group, indicating that GANT61 promoted endometrial repair and ameliorated endometrial fibrosis. Additionally, the effects of GANT61 on *murine* IUA were dose-dependent (Supplementary Fig. 4A–D). Furthermore, combined CQ treatment partially counteracted the effects of GANT61, as indicated by significant changes in *α*SMA (Fig. 4d, e) and collagen I (Fig. 4c, h, i) expression and the trend changes in endometrial thickness (Fig. 4b) and Masson staining (Fig. 4f, g). However, CQ alone had no significant effects on endometrial thickness, *α*SMA, or Masson or collagen I staining (Fig. 4b–i). These data suggest that SHH pathway inhibition mitigated fibrosis, while autophagy blockage blunted that fibrosis alleviation in a *murine* IUA model.

### Constitutive activation of SMO in the endometrial stroma exacerbates fibrosis in the IUA model and is alleviated by the autophagy inducer rapamycin

**Fig. 5:**
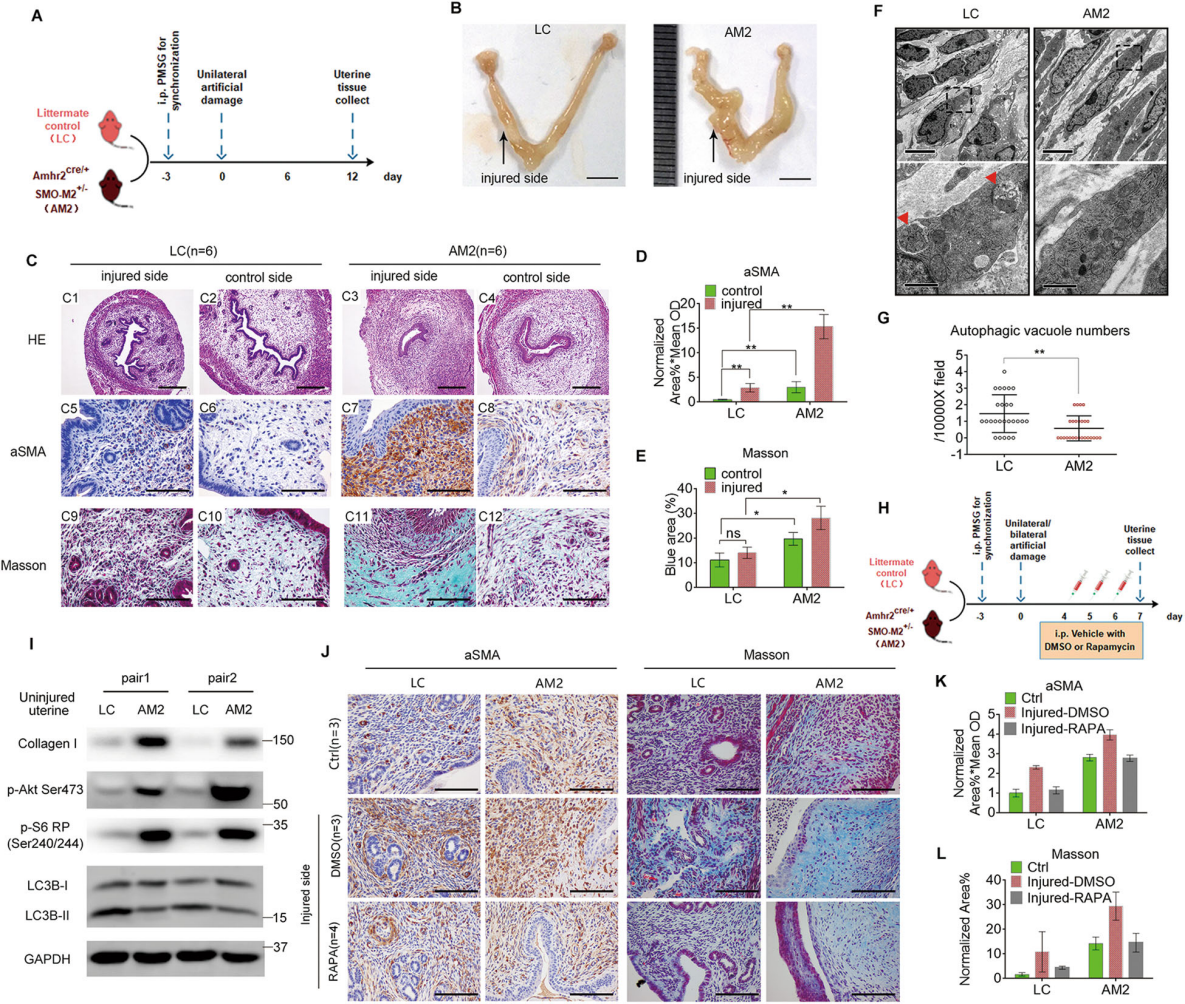
Constitutive activation of SMO in the endometrial stroma exacerbates fibrosis in the IUA model and was alleviated by the autophagy inducer rapamycin. **a** Schematic diagram of establishing the IUA model on *Amhr2*^*cre/+*^*R26-SmoM2*^*+/−*^ (AM2) *mouse* with endometrial stroma-specific constitutive activation of SMO and littermate control (LC) mouse. Uterine tissues were collected on the 12th day. **b** Representative gross uterine photographs of the AM2 and LC IUA models. Scale bars, 5 mm. **c** Representative H&E staining (C1–C4), *α*SMA IHC staining (C5–C8), and Masson staining (C9-C12) images of injured and control side uterine transverse sections from LC (*n* = 6) and AM2 (*n* = 6) *mice*. Scale bars, 20 μm and 100 μm (C1-C4, C5-C12, respectively). **d** Quantitative analysis of *α*SMA IHC staining as determined by the mean OD × %positive area in groups treated as described in C5-C6, by ImageJ. **e** Quantitative analysis of the blue area of Masson staining as determined by the %area in groups treated as described in C9–C12, by ImageJ. **f** Representative TEM images showing endometrial stromal cells from LC and AM2 control sides of the uterus. Pictures in the bottom panel are relative enlargements of the dotted box on the top. Scale bars, 5 μm and 1 μm (top and bottom, respectively). Red triangles indicate autophagic vacuoles. **g** Quantification of autophagic vacuoles per 10,000X field in **f**. **h** Schematic diagram of the detailed treatments with rapamycin (RAPA, 2 mg/kg/day, for 3 days) in a murine IUA model of AM2 and LC mice. Uterine tissues were collected on the 7th day. **i** A section of uterine lysate from AM2 and LC mice was collected for immunoblot analysis of collagen I, p-Akt S473, p-S6 RP Ser240/244 (a substrate of p-p70S6K), LC3B and GAPDH expression levels. **j** Representative *α*SMA IHC staining and Masson staining images of longitudinal uterine sections from LC and AM2 mice in the three groups: Ctrl represents uninjured uterus (*n* = 3); DMSO represents injured uterus with DMSO treatment (*n* = 3); and RAPA represents injured uterus with RAPA treatment (*n* = 4). **k**, **l** Quantitative analysis of *α*SMA IHC staining (**k**) as determined by mean OD × %area and blue area of Masson staining (**l**) as determined by the %area in groups treated as described in (**j**) by ImageJ. Data show mean ± S.E. Statistical analyses were performed by two-way ANOVA plus Bonferroni’s multiple comparisons test (based on matching design; **d, e**). **p < 0.01; *p < 0.05; ns not significant.

To determine the specific impact of the SHH pathway on endometrial stromal cells during fibrosis in vivo, we generated an *Amhr2*^*cre/+*^*R26-SmoM2*^*+/−*^ (AM2) *mouse*, with conditional expression of the SMOM2 protein mutant in the endometrial stroma, to establish an IUA model. A schematic diagram is shown in Fig. 5a. Primary *murine* endometrial stromal cells isolated from AM2 *mouse* showed much higher HH target gene expression than cells from littermate control (LC) as indicated by reverse transcription-quantitative PCR (qRT-PCR; Supplementary Fig. 4E), verifying activation of the HH pathway in endometrial stromal cells.

The AM2 *murine* uterus appeared stiff and grossly hypertrophic (Fig. 5b) and exhibited spindle-shaped myofibroblast-like endometrial stromal cells, stratified luminal epithelial cell layers, and rare uterine glands, as indicated by hematoxylin and eosin (H&E) staining (Fig. 5c1–4), compared to those of the LC. In addition, AM2 *murine* endometrial stroma displayed elevated *α*SMA expression in physiological conditions and significantly increased expression in the IUA model compared to the LC model (Fig. 5c5–c8, d). Similar collagen deposition results were indicated by Masson staining (Fig. 5c9–c12, e). Thus, constitutive activation of SMO in endometrial stromal cells tended to generate fibrogenic characteristics and exacerbated fibrosis in the IUA model.

TEM results demonstrated that endometrial stromal cells from AM2 *mouse* showed fewer autophagic vacuoles and exhibited a slim and stretched-out shape, compared to LC *mouse* cells (Fig. 5f, g). In addition, whole uterine lysates from AM2 *mouse* displayed reduced LC3B-II levels and increased collagen I levels, together with increased expression of p-AKT S473 and p-S6 RP Ser240/244, a direct substrate of p-p70S6K (Fig. 5i).

Furthermore, we used the mTOR inhibitor rapamycin to induce autophagy in the LC group and the AM2 IUA model, and an experimental diagram is shown (Fig. 5h). *α*SMA IHC staining (Fig. 5j, k) and Masson staining (Fig. 5j, l) suggested that rapamycin alleviated fibrosis in both the LC group and AM2 IUA model compared with relative DMSO group. However, the short treatment did not fully alleviate fibrosis in the AM2 IUA model.

## Discussion

**Fig. 6:**
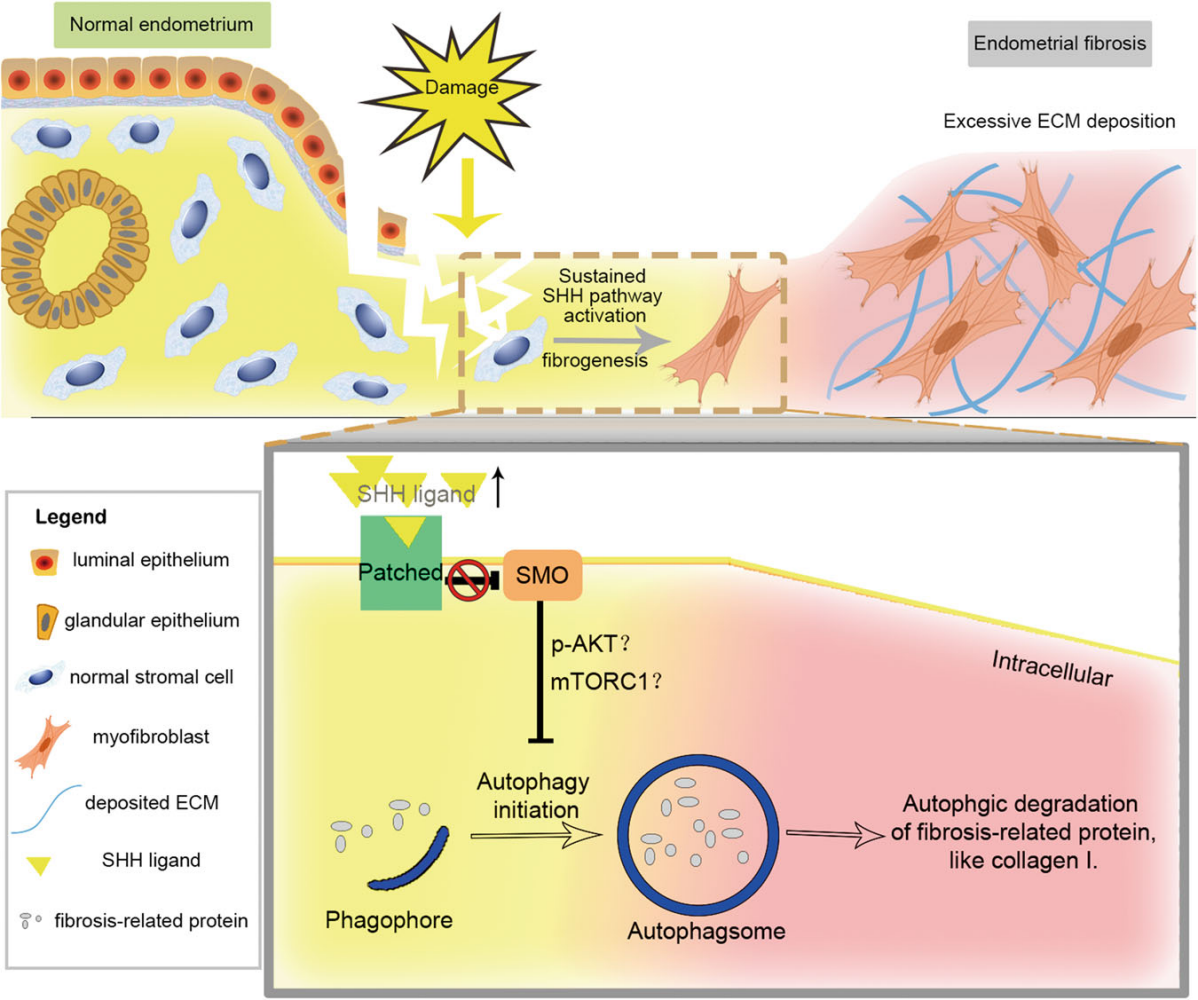
A model of the SHH-inhibited autophagy involved in the pathogenesis of endometrial fibrosis. After damage, elevated SHH ligand expression activates the SHH pathway in endometrial stromal cells, which causes fibrogenesis and drives a stroma-myofibroblast transition if the activation is sustained. For the SHH-driven fibrosis, declined autophagy, which is regulated by SHH pathway and might be mediated by pAKT-mTORC1, influences the degradation of fibrosis-related proteins (like collagen I), contributing to the regulatory mechanism.

The underlying mechanisms of endometrial fibrosis, which leads to infertility and menstrual disorders, require further investigation. We propose for the first time that impaired autophagy participates in SHH pathway-driven endometrial fibrosis (Fig. 6), which is supported by integrated data from an endometrial stromal cell line, *murine* IUA models, endometrial stroma-specific transgenic *mouse*, and clinical IUA samples. Positional analysis showed that autophagy-related markers were depleted in myofibroblasts from fibrotic lesions compared to those of normal endometrial stroma. Mechanistically, in T-HESCs, we demonstrated that SHH pathway affected fibrogenic features by downregulating autophagic degradation of collagen I, which was partially mediated by pAKT-mTORC1. In a *murine* IUA model, SHH pathway inhibition-mediated reduction in endometrial fibrosis could be reversed by autophagy suppression. Sustained activation of the SHH pathway in the endometrial stroma autonomously drove fibrotic features and was associated with decreased autophagy in AM2 transgenic *mouse*. Moreover, AM2 exacerbated fibrosis in the IUA model, which was alleviated by the autophagy inducer rapamycin.

### Sustained SHH pathway activation exacerbates endometrial fibrosis

HH signaling controls embryonic morphogenesis and is essential for adult tissue regeneration. HH signaling is often pathologically activated in multiple organic fibrosis^41^, participating in epithelial-stromal crosstalk and determining stromal cell fate^25,42^. We found aberrant activation of SHH pathway in IUA patients and constitutive activation of SMO in *murine* endometrial stroma exacerbated endometrial fibrosis; inhibition of the SHH pathway exhibited a dose-dependent effect on reducing *murine* fibrosis.

### Autophagy is involved in SHH-induced endometrial fibrosis

The classic mechanism by which SHH pathway regulates fibrosis is through transcriptional regulation of target genes^21,43^, which seems insufficient to account for its fibrotic effects^21^. HH-autophagy regulation is related to apoptosis in hepatocellular carcinoma^29^, osteoblast differentiation^32^, and carotid plaque formation^33^. We demonstrated that impaired autophagy participated in SHH-induced endometrial fibrosis, and that both SHH inhibition and autophagy activation could alleviate fibrogenic progression.

Autophagy is a prerequisite for wound healing^44–46^, and defective autophagy results in abnormal repair with excessive ECM deposition in many tissues, such as the liver^47,48^, kidney^14,49^, and tendon^50^. The autophagic factors LC3B and p62 both displayed a compensatory increase in IUA-detached endometrial stroma and were depleted in myofibroblasts, which is the late-stage form of the stroma. p62 is a well-known selective autophagic cargo receptor that can interact with ubiquitinated substrates via its ubiquitin-associated domain and interact with LC3 via its LC3-interacting region^51^, thus mediating autophagic degradation of ubiquitinated substrates. Analogous with our clinical findings, during the progression of muscular dystrophy in the mdx *mouse* model, autophagy-related molecules (including *Map1lc3b*, *Sqstm1/p62*, and *Becn1*) were increased at the early stage (1.5 months) and progressively reduced at later stages (5, 8, and 12 months) in muscles, and this variation was closely related to the regenerative capacity of satellite cells^52^. Another study indicated that reduced p62 expression was associated with defective autophagy in vascular smooth muscle cells from mitochondrial regulator *Ppargc1a*^*−/−*^ *mouse*^53^. Thus we inferred that autophagy of endometrial stromal cells was initially induced but slowed during endometrial fibrosis progression. However, the role of p62 in the progression of endometrial fibrosis is still under investigation.

We found modulation of that collagen I degradation was involved in the SHH-autophagy-endometrial fibrosis axis. collagen I is the primary structural component of the ECM and provides tensile strength through fibrils^54^, contributing to ECM stiffness and exacerbating fibrosis^5^. Consistent with our results, collagen I could be degraded via autophagy in primary *mouse* mesangial cells^55^ and *human* amnion fibroblasts^56^. Moreover, natural aggregated procollagen^55^, misfolded procollagens^57^ caused by the *Hsp47* disruption, and even internalized collagen from the ECM^58^ can undergo intracellular degradation via autophagy, suggesting a crucial role of autophagy in collagen homeostasis. However, we still have no conclusive evidence to explain why autophagy can reverse SHH pathway-regulated *α*SMA expression in animal experiments.

In addition, the SHH-induced fibrosis abrogation by autophagy was incomplete, indicating that other mechanisms are involved in SHH-induced fibrosis, such as direct transcriptional regulation and the complex interplay with other signaling pathways, such as Hippo, Wnt, and TGF*β*1^25,27,59^ signalings, but these are not included in the scope of this paper.

### SHH pathway regulates autophagy in endometrial stromal cells

Existing evidence indicates that autophagy could be modulated by HH signaling; however, conclusions remain controversial. In our study, SHH signaling suppressed autophagy initiation in endometrial stromal cells (Fig. 2c–k) without significant alterations in cell proliferation, apoptosis, and the cell cycle (Supplementary Fig. 2), avoiding secondary reactions to autophagy caused by other SHH-induced changes in physiological activity. Slight increases in mRNA levels of autophagy-related genes (including *MAP1LC3B*, *ATG5*, and *BECN1*) after GANT61 treatment could also be explained as a compensatory increase due to the promotion of autophagy initiation (Supplementary Fig. 1G). Accordingly, in human hepatocellular carcinoma cells, inhibition of HH signaling induces autophagy, which is associated with the activation of beclin 1^29^, a pivotal component of the autophagy-initiating complex. In HeLa cells and *mouse* embryonic fibroblasts, HH/GLI2 pathway inhibits autophagosome synthesis, and constitutively repressed orthologous Ci expression in *drosophila* induces autophagosome formation^31^. Although SHH promotes autophagy in cultured hippocampal neurons^34^, we think it is due to tissue-specific responsiveness to SHH pathway and the complex transcriptional functions of GLI1-3. Therefore, it is important to study how SHH regulates autophagy in a constant context.

Our study indicated that the AKT-mTOR axis mediated SHH-reduced autophagy initiation in T-HESCs (Fig. 2k, l). Activated AKT boosts TSC2 phosphorylation, which could reduce TSC1/2 complex formation^60^, consequently activating the central autophagic inhibitor mTORC1^61^. mTORC1 serves as a nutrient sensor, integrating multiple upstream signals and transferring information to the autophagy machinery^62^. SHH pathway inhibition significantly reduced mTORC1 activity in parallel with autophagy activation. Among the upstream signals of mTOR (p-AKT, p-AMPK*α*, p-ERK, and p-p38)^63–65^, p-AKT Ser473 likely mediates SHH-autophagy regulation, which was also supported by AM2 *mouse* data.

## Limitations

There are some questions that require further investigation. For instance, we have not definitively identified which autophagy-related genes are regulated by SHH pathway effectors. Additionally, there might be other potential fibrotic markers involved in SHH-autophagy-fibrosis regulation, such as other ECM proteins, cell cycle-related proteins, and fibrogenic factors, which require proteomics comparisons to evaluate and explain autophagy-mediated regulation of *α*SMA. Additionally, how SHH-modulated autophagy affects female pregnancy by affecting the endometrium remains to be studied.

## Material and methods

### Human endometrium samples

From 2018 to 2019, proliferative phase of endometrial fibrous lesions in 16 IUA patients (moderate and severe IUA patients required hysteroscopic separation) and relevantly normal endometria in 10 healthy volunteers (sterile women with male factor or tubal obstruction who required hysteroscopic examination) were sampled at the Sir Run Run Shaw Hospital, Zhejiang University Medical College. The diagnosis of IUA or a normal endometrium was made by an experienced gynecologist based on the hysteroscopic view. The prognostic classification and adhesion scoring of IUA was based on the American Fertility Society Classification of 1988^66^. All cases met the general inclusion criteria: age 20–40 years old, BMI 18.5–24 kg/m^2^, and no severe or systemic diseases. General information for the two groups is available (Supplementary Table S1). Samples were fixed in 4% polyformaldehyde for IHC analysis. For protein lysate extraction, samples were preserved at −80 °C after being frozen in liquid nitrogen. All tissues collected in this study, which remained after pathological diagnosis, were obtained with written informed consent from the patients. This study, including the procedures, was approved by the Ethics Committee of Sir Run Run Shaw Hospital, Zhejiang University Medical College, China (No: 20180226-62).

### Mice

Female 7-week-old ICR strain *mice* were purchased from the Beijing SPF Biotechnology Corporation, Ltd. *R26-SmoM2 mouse* strains were kindly provided by the laboratory of Ximei Wu (Zhejiang University, Hangzhou, China) and were generated as previously described^67,68^. *Amhr2*^*cre/+*^ mouse strains were kindly provided by the laboratory of Jing Li (Nanjing Medical University, Nanjing, China), were generated as previously described and expressed Cre recombinase specifically in the stroma of the uterus^69^. These mice were mated to obtain *Amhr2*^*cre/+*^ *SmoM2*^*+/−*^ mice and were genotyped from tail DNA using protocols from the Jackson Laboratory. The sequences of the corresponding PCR primers are listed in Supplementary Table S2. The *R26-SmoM2* mouse line was engineered with an inserted *SmoM2* gene blocked by a loxP-flanked stop signal. Expression of Cre recombinase in cells released the inhibition of *SmoM2* gene expression. The SMOM2 protein possessed a point mutation that could prevent Patched-mediated inhibition due to continuous activation of the HH pathway. Therefore, female *Amhr2*^*cre/+*^ *SmoM2*^*+/−*^ (AM2) mice were generated as a group with persistently activated Hh signaling in the endometrial stroma. Littermates with other genotypes, including wild-type, *Amhr2*^*cre/+*^ *SmoM2*^*−/−*^, *Amhr2*^*+/+*^ *SmoM2*^*+/−*^, were grouped as the controls. Considering the influence of pregnancy on IUA, we used virgin 6–9-week-old mice to establish an IUA model.

All animals were housed and bred in the Animal Facility of Sir Run Run Shaw Hospital, Zhejiang University according to the institutional guidelines for laboratory animals, and the animal treatment protocol was approved by the Zhejiang University Institutional Animal Care and Use Committee (No: 12146).

### Murine IUA model establishment and drug treatment

The menstrual cycles of all candidate female mice were synchronized by intraperitoneal administration of pregnant mare serum gonadotrophin (PMSG, 5 IU/20 g)^70^. Three days later, a short stumpy uterus made it easy to establish a murine IUA model by electrocoagulation, which was marked as day 0, as previously described^71^. Briefly, the mice were anesthetized by intraperitoneal injection of avertin (0.2 ml/10 g), and abdominal skin preparation was conducted. After opening the abdominal cavity to expose the uterus, a small incision was made at the uterine horn, and then a copper wire (1 mm diameter, only the top of the wire was conductive) wrapped around the working electrode was inserted into uterine lumen through the incision. From near the cervix to the incision in the uterus, electrocoagulation was conducted equally to induce endometrial injury with 0.5 Watts for 2 s. Finally, the uterus was reset, and the abdominal cavity was closed. All surgical procedures were performed under sterile conditions.

In the GANT61 and CQ drug experiments, all unilateral injury ICR mice were randomly divided into four groups, and each group contained five mice. Independent assortment the drug treatments was as follows: vehicle; GANT61 (5 mg/kg/day); CQ (20 mg/kg/day); and GANT61 (5 mg/kg/day) combined with CQ (20 mg/kg/day). According to the manufacturer’s instructions, GANT61 was dissolved in ethanol, and CQ was dissolved in sterilized saline water. Then, we mixed the substances thoroughly according to the formula: 5% ethanol (with or without GANT61), 40% PEG400, 5% Tween 80, and 50% saline water (with or without CQ). All solutions were freshly prepared and used for intraperitoneal administration on days 4, 5, and 6. On day 7, the mice were anesthetized for uterine collection.

In LC and AM2 transgenic mouse experiments, unilateral injury was performed to explore the difference in fibrosis between LC and AM2 uteri under natural or injured conditions. To find a significant difference, we selected a longer time, day 12, when most LC IUA models had healed, to collect the tissues. For administration to AM2 mice, rapamycin was dissolved in dimethyl sulfoxide (DMSO) and further prepared using the following formula: 5% DMSO (with or without rapamycin), 30% PEG400, 5% Tween 80, and 60% saline. Rapamycin was used at 2 mg/kg/day, and these solutions were all freshly prepared and used for intraperitoneal administration on days 4, 5, and 6. On day 7, the mice were anesthetized for uterine collection.

All processes, including model establishment, grouping, treatments, and sample collection, followed the principles of random and blind analyses.

### Cell lines, primary mouse endometrial stromal cell isolation, chemicals, and plasmids

T-HESCs (*human* telomerase reverse transcriptase-immortalized endometrial stromal cells, ATCC, CRL-4003) were cultured in DMEM/F12 medium without phenol red (Sigma) supplemented with 10% charcoal/dextran-treated fetal bovine serum (HyClone), 1.5 g/L sodium bicarbonate, 1% ITS (Gibco), and 500 ng/mL puromycin. This cell line was authenticated by morphology and growth trait examination and tested free of mycoplasma contamination.

Primary *mouse* endometrial stromal cells were obtained from three mice/group according to the protocol from JoVE^72^, and total RNA was extracted from primary endometrial stromal cells within 6 h of cell culture to be similar to the in vivo state.

The following chemicals were used in this study: PUR (Selleck, S3042), GANT61 (Selleck, S8075), rapamycin (Sirolimus) (Selleck, S1039), CQ (Sigma, C6628), 3-MA (Selleck, S2767), and SC79 (Selleck, S7863).

The recombinant plasmid p*SHH-*fl (inserted with the full-length open reading frame of the *human SHH* gene) and its vector pCMV were kindly provided by Dr. Hongfeng Ruan of Zhejiang Chinese Medical University. The Luc2p-p62 plasmid was donated by Dr. Chenguang Zhang of Capital Medical University and expressed a recombinant p62/SQSTM1 protein fused with a luciferase variant of Luc2p at the N-terminus that was degraded via autophagy; thus, the luciferase activity of the cell lysates indicates autophagic flux, as previously described^38^.

### Western blot analysis

The membranes were blocked with 5% BSA (for phosphorylated proteins) or 5% skim milk (for other proteins) in Tris-buffered saline containing Tween-20 at room temperature (RT). Then, the membranes were incubated with primary antibodies against GAPDH (1:5000; Proteintech, 60004-1-Ig), LC3B (1:2000; Sigma, L7543), SQSTM1/p62 (1:2000; Abcam, ab56416), SHH (1:1000; Abcam, ab53281), collagen I (1:2000; Abcam, ab138492), *α*SMA (1:10000; Abcam, ab124964), p-p70S6K Thr389 (1:1000; Cell Signaling Technology, 9205), p-AMPK*α*1/2 Thr183/Thr172 (1:2000; Abcam, ab133448), p-ERK1/2 (Thr202/Tyr204) (1:1000; Cell Signaling Technology, 4370), p-p38 Thr180/Tyr182 (1:1000; Cell Signaling Technology, 9215), p-AKT Ser473 (1:1000; Cell Signaling Technology, 4060), p-S6 RP Ser240/244 (1:1000; Cell Signaling Technology, 5364), ATG5 (1:1000; Cell Signaling Technology, 12994), and ATG7 (1:1000; Cell Signaling Technology, 8558) overnight at 4 °C with shaking. The membranes were incubated with HRP-conjugated *goat* anti-*rabbit* IgG (1:5000; BD Pharmingen, 554021), goat anti-*mouse* IgG (1:5000; BD Pharmingen, 554002), or HRP-conjugated *mouse* anti-*rabbit* IgG light chain specific (1:5000; Proteintech, SA00001-7L, specific for SHH protein detection in *human* tissue), preadsorbed *goat* anti-*rabbit* IgG H&L IRDye 800 (1:10000; Abcam, ab216773), or *donkey* anti-*mouse* IgG H&L Alexa Fluor 680 (1:10000; Abcam, ab175774) secondary antibodies for 60 min at RT. Protein expression was detected by an enhanced chemiluminescence kit (Millipore) or an Odyssey Infrared Laser Imaging System (Licor Bioscience, CLX) and was normalized to the expression of GAPDH.

### Immunohistochemistry, Masson’s trichrome staining, and immunofluorescence analysis

#### Immunohistochemistry

Slides of 4% PFA-fixed, paraffin-embedded sections (7 μm) were subjected to IHC analysis. After routine deparaffinization and rehydration, the slides were heat-induced at 100 °C in citrate sodium buffer (10 mM, pH 6.0) for 20 min to retrieve antigens. Subsequently, 3% H_2_O_2_ was used to suppress endogenous peroxidase activity, and blocking buffer (ab64226) was used to reduce nonspecific binding. Antibodies against *α*SMA (1:800; Abcam, ab124964), collagen I (1:100; Abcam, ab34710), SQSTM1/p62 (1:400; MBL International, PM045), and the negative control rabbit IgG were applied in the same manner for each sample. There was no specific staining detected in the negative controls.

#### Masson’s trichrome staining

A Masson’s trichrome staining kit (Solarbio, G1345) was used according to the manufacturer’s instructions. Briefly, sections were fixed with Bouin buffer, nuclei were counterstained blue-brown with Celestine blue and Mayer sumuin solutions, collagenous fibers were stained bright blue with phosphate acid and aniline blue solutions, and other components, such as muscle fibers, keratin, and erythrocytes, were dyed red with Ponceau and Fuchsin solutions. Then, the slides were dehydrated and mounted for analysis. To quantify blue-stained fibers in each group, representative slides per uterine side were chosen, and at least four pictures of a high-power field were captured for each slide. The blue area percentages were calculated by ImageJ software with the plugin IHC Tool Box.

#### Immunofluorescence

About 1 × 10^4^ cells were plated on 14 mm diameter slides, and cultured for 24 h followed by drugs treatments. Then cells were were fixed with 4% PFA for 15 min, permeabilized with 0.01% Triton X-100, and blocked with 5% BSA for 1 h, followed by overnight incubation with primary antibody (collagen I 1:100, Abcam, ab34710 for cells; LC3B 1:100, Sigma, L7543 for tissue sections) or isotype control IgG overnight at 4 °C. DyLight488 *goat* anti-*rabbit* IgG (1:200; MultiSciences, GAR4882) was used as the secondary antibody, and DAPI was used for nuclear counterstaining. For tissue section immunofluorescence, slides were incubated in 0.5% Chicago Sky Blue 6B solution (Apexbio, B6473) for 10 min to reduce spontaneous green fluorescence after routine deparaffinization and rehydration, and then the samples underwent antigen retrieval and further steps as previously described. Fluorescent images were captured by confocal microscopy (Olympus FV3000), and the images that needed to be compared were captured under uniform excitation and imaging conditions.

### RNA isolation and reverse transcription-quantitative PCR

Total RNA was extracted from cells by TRIzol reagent (Invitrogen) according to the manufacturer’s protocol. Purified total RNA was cleaned of genomic DNA and reverse-transcribed into cDNA using random primers with a HiScript® II Q RT SuperMix for qPCR (+g DNA wiper) kit (Vazyme, R223-01) under reaction conditions of 50 °C for 15 min and 85 °C for 5 s. Then, qRT-PCR was performed using a SYBR green PCR kit and a real-time PCR detection system (Roche, LightCycler 480). The amplification program was 40 cycles of the following: 95 °C for 15 s, 58 °C for 30 s, and 72 °C for 30 s.

The relative expression of the selected genes was normalized to that of the reference gene set (GAPDH, β-actin, and 18 s) and quantified using the 2^−ddCT^ method unless otherwise stated. The sequences of the corresponding qRT-PCR primers are listed in Supplementary Table S2.

### Electron microscopy

Freshly collected cell masses or tissue samples were fixed with 2.5% glutaraldehyde for 24 h, followed by postfixation with 1% osmium tetroxide for 1 h. Then, the samples were stained with 2% uranium acetate for 30 min, dehydrated with gradient ethanol, and sequentially embedded with 50 and 100% eponate 12 medium. The endometrial stroma of tissue was located through the luminal epithelium, cut with a diamond knife into thin sections (150 nm), and analyzed using a Tecnai T10 100 kV electron microscope (Philips). Sample disposal and sectioning were performed by specialists at the Center of Cryo-Electron Microscopy of Zhejiang University. Autophagic vacuoles per 10,000X field were counted and at least 14 images per group from three independent experiments were used for quantitation. Representative autophagic vacuoles in electron microscopic images were marked as previously described^38^.

### Transfection and lentiviral transduction

For plasmid transfection or RNAi experiments, T-HESCs at ~50% confluence were transfected with plasmid DNA (1 µg) or small interfering RNAs (50 nmol) by Lipofectamine 3000 reagent (Invitrogen) according to the manufacturer’s instructions. Approximately 24–36 h later, cells were collected for efficiency tests or were further treated. The targeting sequences of human *ATG5* and *ATG7* (RuiBo Biological Technology Co., Ltd.) are listed in Supplementary Table S2.

Adenovirus expressing an mCherry-GFP-LC3B fusion protein (Ad-mCherry-GFP-LC3B) was purchased from (Beyotime, C3011) and used to monitor autophagy as described^37^. After the cells were evenly attached to the slides, adenovirus diluted in antibiotic-free medium was added to each well and incubated for 24 h. Then, infected cells were subjected to different treatments, and treated cells were fixed with methanol, counterstained with DAPI, and analyzed by confocal microscopy (Olympus FV3000). For each group, at least 15 images from three independent experiments at ×600 magnification in Z axis mode were taken, and were analyzed by ImageJ 1.52 software (National Institutes of Health, USA) by counting the yellow vesicles (GFP + mCherry+ puncta, representing autophagosomes) and red vesicles (GFP-mCherry+ puncta, representing autolysosomes) in individual cells.

### Cell Counting Kit-8 (CCK-8) assay, Annexin V-phycoerythrin/7-amino-actinomycin D (Annexin V-PE/7-AAD) assay, and cell cycle assay

CCK-8 (Dojindo, Japan) assays were performed at 0, 1, 2, 3, and 4 days after drug treatment. A total of 1000 cells were cultured per well of a 96-well plate for 24 h, different drugs or vehicles were added to the culture medium according to the experimental design, and the treatments were refreshed every 2 days. For measurement, 100 μl of culture medium containing 10% CCK-8 was added per well and incubated in the dark for 2 h. Then, the OD values at 450 nm were measured by a microplate reader (Thermo Scientific Multiskan GO). Each treatment included five wells, and three independent tests were performed.

To analyze the effects of the drugs on cell apoptosis, an Annexin V-PE and 7-AAD apoptosis detection kit (BD Biosciences, 559763) was used 24 h after drug treatment according to the manufacturer’s protocol. Briefly, intact treated cells were collected, washed twice with cold PBS, and then stained with Annexin V-PE/7-AAD for 15 min in the dark at a concentration of 1 × 10^6^ cells/ml. Experimental control groups included unstained, PE-stained, 7-AAD-stained, and positive dead cells. The cells were detected by a BD LSRFortessa^TM^ flow cytometer and analyzed by FlowJo X software (Becton, Dickinson & Company, San Jose, CA, USA)^65^.

To analyze the effects of drugs on the cell cycle, propidium iodide (Invitrogen, P3566) was used to dye the DNA at 24 h after drug treatment according to the manufacturer’s protocol. Briefly, intact treated cells were collected, fixed with cold 70% ethanol at −20 °C overnight, and then incubated in a solution containing 50 μg/ml PI, 100 ug/ml ribonuclease A, and 0.2% Triton X-100 at 4 °C in the dark for 30 min. The cells were detected by a CytoFLEX flow cytometer and analyzed by ModFit LT5.0 software (Verity Software House, Topsham, ME, USA).

### Statistical analysis

GraphPad Prism 6 (GraphPad, San Diego, California, USA) was used for data arrangement and statistical analysis. The data presented in this study are the mean ± SD if not otherwise stated. KS normality test was used for Gaussian distribution examination and Bartlett’s test was used to estimate statistical difference of standard deviations between groups. Statistical analyses were performed by two-sided Mann–Whitney *U*-test comparisons of two groups with abnormal distribution. For animal experiments of the unilateral IUA model, two-way ANOVA plus Bonferroni’s multiple comparisons test (based on matching design) was applied. After normalization of changed values between the corresponding injured and control groups (to eliminate individual differences), the Kruskal–Wallis test plus Dunn’s multiple comparisons test was used to compare the variance between groups. A value of *p* < 0.05 was considered significant.

## Supplementary information

supplementary figure and table legends

Supplementary Figure 1. The gradient drug treatments of T-HESCs.

Supplementary Figure 2. Treatment with 5 μM GANT61 for 24 h had no significant effect on the proliferation and apoptosis of T-HESCs.

Supplementary Figure 3. Important roles of Collagen I in the Hh-autophagy-fibrosis regulatory axis.

Supplementary Figure 4. GANT61 reduced endometrial fibrosis in a dose-dependent manner in the murine IUA model.

Supplementary Table S1. Details of women donated with NE or IUA samples

Supplementary Table S2. Primer squences List of RT-qPCR, genetyping mice and siRNA target squences of corresponding genes

## References

[1] Evans J (2016). Fertile ground: human endometrial programming and lessons in health and disease. Nat. Rev. Endocrinol..

[2] Strowitzki T, Germeyer A, Popovici R, von Wolff M (2006). The human endometrium as a fertility-determining factor. Hum. Reprod. Update.

[3] Yu D, Wong YM, Cheong Y, Xia E, Li TC (2008). Asherman syndrome–one century later. Fertil. Steril..

[4] Deans R, Abbott J (2010). Review of intrauterine adhesions. J. Minim. Invasive Gynecol..

[5] Hinz B (2012). Recent developments in myofibroblast biology: paradigms for connective tissue remodeling. Am. J. Pathol..

[6] Darby IA, Zakuan N, Billet F, Desmouliere A (2016). The myofibroblast, a key cell in normal and pathological tissue repair. Cell Mol. Life Sci..

[7] Kramann R, Schneider RK (2018). The identification of fibrosis-driving myofibroblast precursors reveals new therapeutic avenues in myelofibrosis. Blood.

[8] Kanisicak O (2016). Genetic lineage tracing defines myofibroblast origin and function in the injured heart. Nat. Commun..

[9] LeBleu VS (2013). Origin and function of myofibroblasts in kidney fibrosis. Nat. Med..

[10] Bento CF (2016). Mammalian autophagy: how does it work?. Annu. Rev. Biochem..

[11] Xie Z, Klionsky DJ (2007). Autophagosome formation: core machinery and adaptations. Nat. Cell Biol..

[12] Hurley JH, Young LN (2017). Mechanisms of autophagy initiation. Annu. Rev. Biochem..

[13] Dikic I, Elazar Z (2018). Mechanism and medical implications of mammalian autophagy. Nat. Rev. Mol. Cell Biol..

[14] Li H (2016). Atg5-mediated autophagy deficiency in proximal tubules promotes cell cycle G2/M arrest and renal fibrosis. Autophagy.

[15] Xu G (2016). Defects in MAP1S-mediated autophagy turnover of fibronectin cause renal fibrosis. Aging.

[16] Cabrera S (2015). Essential role for the ATG4B protease and autophagy in bleomycin-induced pulmonary fibrosis. Autophagy.

[17] Petrov K, Wierbowski BM, Salic A (2017). Sending and receiving hedgehog signals. Annu. Rev. Cell Dev. Biol..

[18] Bangs F, Anderson KV (2017). Primary cilia and mammalian hedgehog signaling. Cold Spring Harb. Perspect. Biol..

[19] Rimkus TK, Carpenter RL, Qasem S, Chan M, Lo HW (2016). Targeting the sonic hedgehog signaling pathway: review of smoothened and GLI inhibitors. Cancers.

[20] Katoh Y, Katoh M (2009). Hedgehog target genes: mechanisms of carcinogenesis induced by aberrant hedgehog signaling activation. Curr. Mol. Med..

[21] Ding H (2012). Sonic hedgehog signaling mediates epithelial-mesenchymal communication and promotes renal fibrosis. J. Am. Soc. Nephrol..

[22] Zhou D (2014). Sonic hedgehog is a novel tubule-derived growth factor for interstitial fibroblasts after kidney injury. J. Am. Soc. Nephrol..

[23] Kramann R (2015). Perivascular Gli1+ progenitors are key contributors to injury-induced organ fibrosis. Cell Stem Cell.

[24] Lim CH (2018). Hedgehog stimulates hair follicle neogenesis by creating inductive dermis during murine skin wound healing. Nat. Commun..

[25] Du K (2018). Hedgehog-YAP signaling pathway regulates glutaminolysis to control activation of hepatic stellate cells. Gastroenterology.

[26] Wang X (2016). Hepatocyte TAZ/WWTR1 promotes inflammation and fibrosis in nonalcoholic steatohepatitis. Cell Metab..

[27] Liang R (2017). The transcription factor GLI2 as a downstream mediator of transforming growth factor-beta-induced fibroblast activation in SSc. Ann. Rheum. Dis..

[28] Lin X (2018). Endometrial stem cell-derived granulocyte-colony stimulating factor attenuates endometrial fibrosis via sonic hedgehog transcriptional activator Gli2. Biol. Reprod..

[29] Wang Y, Han C, Lu L, Magliato S, Wu T (2013). Hedgehog signaling pathway regulates autophagy in human hepatocellular carcinoma cells. Hepatology.

[30] Lo Re AE (2012). Novel AKT1-GLI3-VMP1 pathway mediates KRAS oncogene-induced autophagy in cancer cells. J. Biol. Chem..

[31] Jimenez-Sanchez M (2012). The Hedgehog signalling pathway regulates autophagy. Nat. Commun..

[32] Hu Z., Chen B., Zhao Q. Hedgehog signaling regulates osteoblast differentiation in zebrafish larvae through modulation of autophagy. *Biol. Open* **8**, bio040840 (2019). 10.1242/bio.040840 PMC6550075 30992325

[33] Li H (2012). Sonic hedgehog promotes autophagy of vascular smooth muscle cells. Am. J. Physiol. Heart Circ. Physiol..

[34] Petralia RS (2013). Sonic hedgehog promotes autophagy in hippocampal neurons. Biol. Open.

[35] Klionsky DJ (2016). Guidelines for the use and interpretation of assays for monitoring autophagy. Autophagy.

[36] Seglen PO, Gordon PB (1982). 3-Methyladenine: specific inhibitor of autophagic/lysosomal protein degradation in isolated rat hepatocytes. Proc. Natl Acad. Sci. USA.

[37] Peng Y (2017). M4IDP, a zoledronic acid derivative, induces G1 arrest, apoptosis and autophagy in HCT116 colon carcinoma cells via blocking PI3K/Akt/mTOR pathway. Life Sci..

[38] Min Z (2018). Monitoring autophagic flux using p62/SQSTM1 based luciferase reporters in glioma cells. Exp. Cell Res..

[39] Araki S (2015). Sirt7 contributes to myocardial tissue repair by maintaining transforming growth factor-beta signaling pathway. Circulation.

[40] Liang C (2020). TGFB1-induced autophagy affects the pattern of pancreatic cancer progression in distinct ways depending on SMAD4 status. Autophagy.

[41] Distler JHW (2019). Shared and distinct mechanisms of fibrosis. Nat. Rev. Rheumatol..

[42] Kramann R (2015). Pharmacological GLI2 inhibition prevents myofibroblast cell-cycle progression and reduces kidney fibrosis. J. Clin. Invest..

[43] Hu B (2015). Reemergence of hedgehog mediates epithelial-mesenchymal crosstalk in pulmonary fibrosis. Am. J. Respir. Cell Mol. Biol..

[44] An Y (2018). Autophagy promotes MSC-mediated vascularization in cutaneous wound healing via regulation of VEGF secretion. Cell Death Dis..

[45] Xu Z, Klionsky DJ (2016). Autophagy promotes cell motility by driving focal adhesion turnover. Autophagy.

[46] Levin AD (2016). Autophagy contributes to the induction of anti-TNF induced macrophages. J. Crohns Colitis.

[47] Zhang, X.-W. et al. Disrupting the TRIB3-SQSTM1 interaction reduces liver fibrosis by restoring autophagy and suppressing exosome-mediated HSC activation. *Autophagy* **16**, 1–15 (2019). 10.1080/15548627.2019.1635383 PMC7144866 31286822

[48] Ni H-M (2014). Nrf2 promotes the development of fibrosis and tumorigenesis in mice with defective hepatic autophagy. J. Hepatol..

[49] Livingston MJ (2016). Persistent activation of autophagy in kidney tubular cells promotes renal interstitial fibrosis during unilateral ureteral obstruction. Autophagy.

[50] Zheng W, Qian Y, Chen S, Ruan H, Fan C (2018). Rapamycin protects against peritendinous fibrosis through activation of autophagy. Front. Pharmacol..

[51] Pankiv S (2007). p62/SQSTM1 binds directly to Atg8/LC3 to facilitate degradation of ubiquitinated protein aggregates by autophagy. J. Biol. Chem..

[52] Fiacco E (2016). Autophagy regulates satellite cell ability to regenerate normal and dystrophic muscles. Cell Death Differ..

[53] Salazar G. et al. SQSTM1/p62 and PPARGC1A/PGC-1alpha at the interface of autophagy and vascular senescence. *Autophagy* **16**, 1–19 (2019). 10.1080/15548627.2019.1659612 PMC7469683 31441382

[54] Bonnans C, Chou J, Werb Z (2014). Remodelling the extracellular matrix in development and disease. Nat. Rev. Mol. Cell Biol..

[55] Kim SI (2012). Autophagy promotes intracellular degradation of type I collagen induced by transforming growth factor (TGF)-beta1. J. Biol. Chem..

[56] Mi Y (2017). Autophagic degradation of collagen 1A1 by cortisol in human amnion fibroblasts. Endocrinology.

[57] Ishida Y (2009). Autophagic elimination of misfolded procollagen aggregates in the endoplasmic reticulum as a means of cell protection. Mol. Biol. Cell.

[58] Kawano S (2017). Autophagy promotes degradation of internalized collagen and regulates distribution of focal adhesions to suppress cell adhesion. Biol. Open.

[59] Petherick KJ (2013). Autolysosomal beta-catenin degradation regulates Wnt-autophagy-p62 crosstalk. EMBO J..

[60] Manning BD, Tee AR, Logsdon MN, Blenis J, Cantley LC (2002). Identification of the tuberous sclerosis complex-2 tumor suppressor gene product tuberin as a target of the phosphoinositide 3-kinase/akt pathway. Mol. Cell.

[61] Long X, Lin Y, Ortiz-Vega S, Yonezawa K, Avruch J (2005). Rheb binds and regulates the mTOR kinase. Curr. Biol..

[62] Dobrenel T (2016). TOR signaling and nutrient sensing. Annu. Rev. Plant Biol..

[63] He C, Klionsky DJ (2009). Regulation mechanisms and signaling pathways of autophagy. Annu. Rev. Genet..

[64] Bond P (2016). Regulation of mTORC1 by growth factors, energy status, amino acids and mechanical stimuli at a glance. J. Int. Soc. Sports Nutr..

[65] He D (2020). Gut stem cell aging is driven by mTORC1 via a p38 MAPK-p53 pathway. Nat. Commun..

[66] The American Fertility Society. Classifications of adnexal adhesions, distal tubal occlusion, tubal occlusion secondary to tubal ligation, tubal pregnancies, mullerian anomalies and intrauterine adhesions. *Fertil. Steril.* **49**, 944–955 (1988). 10.1016/s0015-0282(16)59942-7 3371491

[67] Jeong J, Mao J, Tenzen T, Kottmann AH, McMahon AP (2004). Hedgehog signaling in the neural crest cells regulates the patterning and growth of facial primordia. Genes Dev..

[68] Zhang XM, Ramalho-Santos M, McMahon AP (2001). Smoothened mutants reveal redundant roles for Shh and Ihh signaling including regulation of L/R symmetry by the mouse node. Cell.

[69] Jamin SP, Arango NA, Mishina Y, Hanks MC, Behringer RR (2002). Requirement of Bmpr1a for Müllerian duct regression during male sexual development. Nat. Genet..

[70] Zhou T, Hu M, Pearlman A, Rohan LC (2016). Expression, regulation, and function of drug transporters in cervicovaginal tissues of a mouse model used for microbicide testing. Biochem. Pharmacol..

[71] Zhang Y (2016). Endometrial stem cells repair injured endometrium and induce angiogenesis via AKT and ERK pathways. Reproduction.

[72] De Clercq K., Hennes A., Vriens J. Isolation of mouse endometrial epithelial and stromal cells for in vitro decidualization. *J. Vis. Exp.* **2**, 55168 (2017). 10.3791/55168 PMC5408775 28287563

