# Correction: Overactivated sonic hedgehog signaling aggravates intrauterine adhesion via inhibiting autophagy in endometrial stromal cells

**DOI:** 10.1038/s41419-020-03128-y

**Published:** 2020-10-30

**Authors:** Cheng Wei, Yibin Pan, Yinli Zhang, Yongdong Dai, Lingling Jiang, Libing Shi, Weijie Yang, Shiqian Xu, Yingyi Zhang, Wenzhi Xu, Yanling Zhang, Xiaona Lin, Songying Zhang

**Affiliations:** 1 Assisted Reproduction Unit, Department of Obstetrics and Gynecology, Sir Run Run Shaw Hospital, School of Medicine, Zhejiang University, Hangzhou, 310016 China; 2 Key Laboratory of Reproductive Dysfunction Management of Zhejiang Province, No. 3 Qingchun East Road, Jianggan District, Hangzhou, 310016 China

**Keywords:** Autophagy, Reproductive disorders, Pathogenesis

Correction to: *Cell Death and Disease*

10.1038/s41419-020-02956-2 published online 15 September 2020

Following publication of the original article, the authors wished to add the following funding source to the Acknowledgements section:

This research was supported by grants from National Key Research and Development Program of China (2018YFC1004800), National Natural Science Foundation of China (81871209 and 81601308), Provincial and Ministerial Co Construction Project of Zhejiang Province (WKJ-ZJ-1721), Natural Science Foundation of Zhejiang Province (Y19H040065, LQ20H040004, and LQ18H160005), Zhejiang Medical Science and Technology Project (2018RC009), and The Chinese Medical Association Clinical Doctors Scientific Research Fund (18010280757).

The authors apologise for this omission. Both the PDF and HTML versions of the Article have been updated accordingly.

