# Supplementary material for: Overactivated sonic hedgehog signaling aggravates intrauterine adhesion via inhibiting autophagy in endometrial stromal cells
Source: Cell Death Dis. 2020 Sep 15;11(9):755. doi: 10.1038/s41419-020-02956-2 (PMC7492405; doi:10.1038/s41419-020-02956-2)
Supplement: Supplementary file 1 — supplementary figure and table legends [file 41419_2020_2956_MOESM1_ESM.docx]

**Supplementary Figure 1. The gradient drug treatments of T-HESCs.**

**A**, Representative IHC staining of PTCH1 receptor in the human endometrial stromal cell. Scale bar, 200μm. **B,** Relative mRNA level of *SHH* and hedgehog pathway target genes (*GLI1, PTCH1*) of T-HESCs after transfections of vector pCMV or p*SHH*fl for 48h. Data show mean ± S.D. **C.D**,T-HESC cells were treated with gradient doses of SMO agonist (**C,**PUR), GLI inhibitor (**D,**GANT61) for 24 hours before being collected for immunoblotting analysis against LC3B. **E**, Relative hedgehog pathway target genes(*GLI1, PTCH1*) mRNA level of T-HESCs after being treated with gradient doses of GANT61 or PUR for 24h. Relative expression was calculated using ΔΔCt. Data show mean ± S.D. **F**, T-HESC cells were treated with SC79 (AKT activator) for 30 minutes before immunoblotting analysis of p-AKT Ser473 and p-GSK3*β* Ser9 (substrate of pAKT). **G**, Relative mRNA level of autophagy-related genes (*MAP1LC3B, ATG5, BECN1*) of T-HESCs after gradient GANT61 treatment for 24 hours. Data show mean ± S.D.

**Supplementary Figure 2. Treatment with 5 μM GANT61 for 24 h had no significant effect on the proliferation and apoptosis of T-HESCs.**

**A,** The CCK-8 assay was used to assess the proliferation of T-HESCs that were treated with vehicle or GANT61 (5 μM) for 4 days. Data show mean ± S.E. for three independent experiments. **B. C,** T-HESCs apoptosis was measured by flow cytometric analysis of Annexin V-PE and 7-AAD staining before the cells were treated with vehicle (left) or 5 μM GANT61 (right) for 24 h. Representative density plots (at least 10,000 events) were obtained with FlowJo X software and are displayed in (**B**). Quadrants delimited positive (+) and negative (-) events, and the percentage of events in each quadrant is shown in (**C**). The populations in Q4 (PE−/7AAD−), Q3 (PE+/7AAD−) and Q2 (PE+/7AAD+) correspond to viable cells, early apoptotic cells, and late apoptotic cells, respectively. **D. E**, T-HESC cell cycle was measured by flow cytometric analysis of propidium iodide staining before the cells were treated with vehicle (left) or GANT61 5 μM (right) for 24 h. Representative plots (at least 10,000 events) were analyzed with ModFit LT5.0 software and are displayed in (**E**); the percentage of each cell cycle stage is shown in (**D**). Statistical analyses were performed by two-way ANOVA plus Bonferroni's multiple comparisons test (**A**) compared to the vehicle group. ns, not significant.

**Supplementary Figure 3. Important roles of collagen I in the HH-autophagy-fibrosis regulatory axis.**

**A**, Representative immunoblotting images against LC3B and other ECM molecules in T-HESCs treated with autophagic stimulant (EBSS) for 4h or lysosomal inhibitors (CQ) for 8h, GAPDH was shown in figure 4A. **B,** Representative immunoblotting images against collagen I of T-HESCs treated with PUR for 24h. **C**, Relative *COL1A1* and *COL1A2* mRNA level of T-HESCs treated with/without GANT61 for 24h followed by in the presence or absence of CQ for 4h. Data show mean ± S.E. for three independent experiments.

**Supplementary Figure 4. GANT61 reduced endometrial fibrosis in a dose-dependent manner in the *murine* IUA model.**

**A, B, C,** Representative H&E staining (**A**), *α*SMA IHC staining (**B**),Masson staining (**C**) images of both injured and control side transversal uterine sections. VE, vehicle; G1, GANT61 (1mg/kg/day); G5, GANT61 (5mg/kg/day); G15, GANT61 (15mg/kg/day) for 3 days. Each group included 4 mice. Scale bars, 25μm(**A**) and 100μm (**B, C**). **D,** Thickness of normal endometrium of both control and injured side in 4 groups. Data show mean ± S.E. Statistical analyses were performed by two-way ANOVA plus Bonferroni's multiple comparisons test (based on matching design) comparing to relative control uterine thickness. **E**, Relative hedgehog pathway target genes of *Ptch1* and *Gli1* mRNA level of primary *mouse* endometrial stromal cells isolated from AM2 (n=3) and LC (n=3) mice. Data show mean ± S.D.

**Supplementary Table S1. Details of women donated with NE or IUA samples.**

Definition of abbreviations: NE = normal endometrium. IUA = Intrauterine adhesion. Abortion history is included in all spontaneous or artificial abortion during whole gestation. Adhesion scoring scoring of IUA was based on ‘American fertility society classification 1988’. Statistical analyses were performed by two-sided Mann-Whitney U-test comparsion. Data was presents as mean ± SD; ns, no significance, ** p<0.01.

**Supplementary Table S2. Primer squences List of RT-qPCR, genetyping mice and siRNA target squences of corresponding genes**
