# Supplementary figures and images for: Overactivated sonic hedgehog signaling aggravates intrauterine adhesion via inhibiting autophagy in endometrial stromal cells
Source: Cell Death Dis. 2020 Sep 15;11(9):755. doi: 10.1038/s41419-020-02956-2 (PMC7492405; doi:10.1038/s41419-020-02956-2)

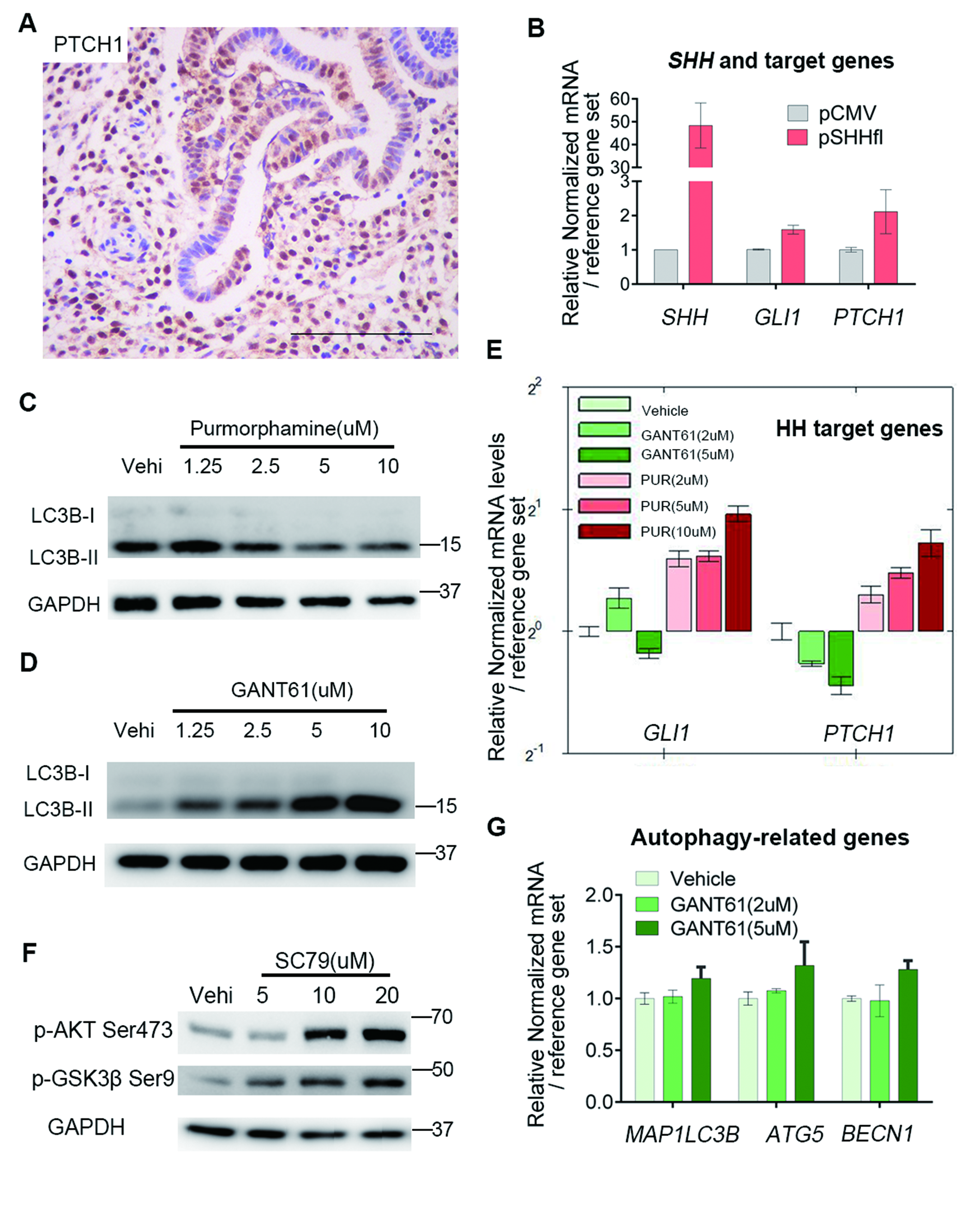

Supplement: Supplementary file 2 — Supplementary Figure 1. The gradient drug treatments of T-HESCs. [file 41419_2020_2956_MOESM2_ESM.tif]

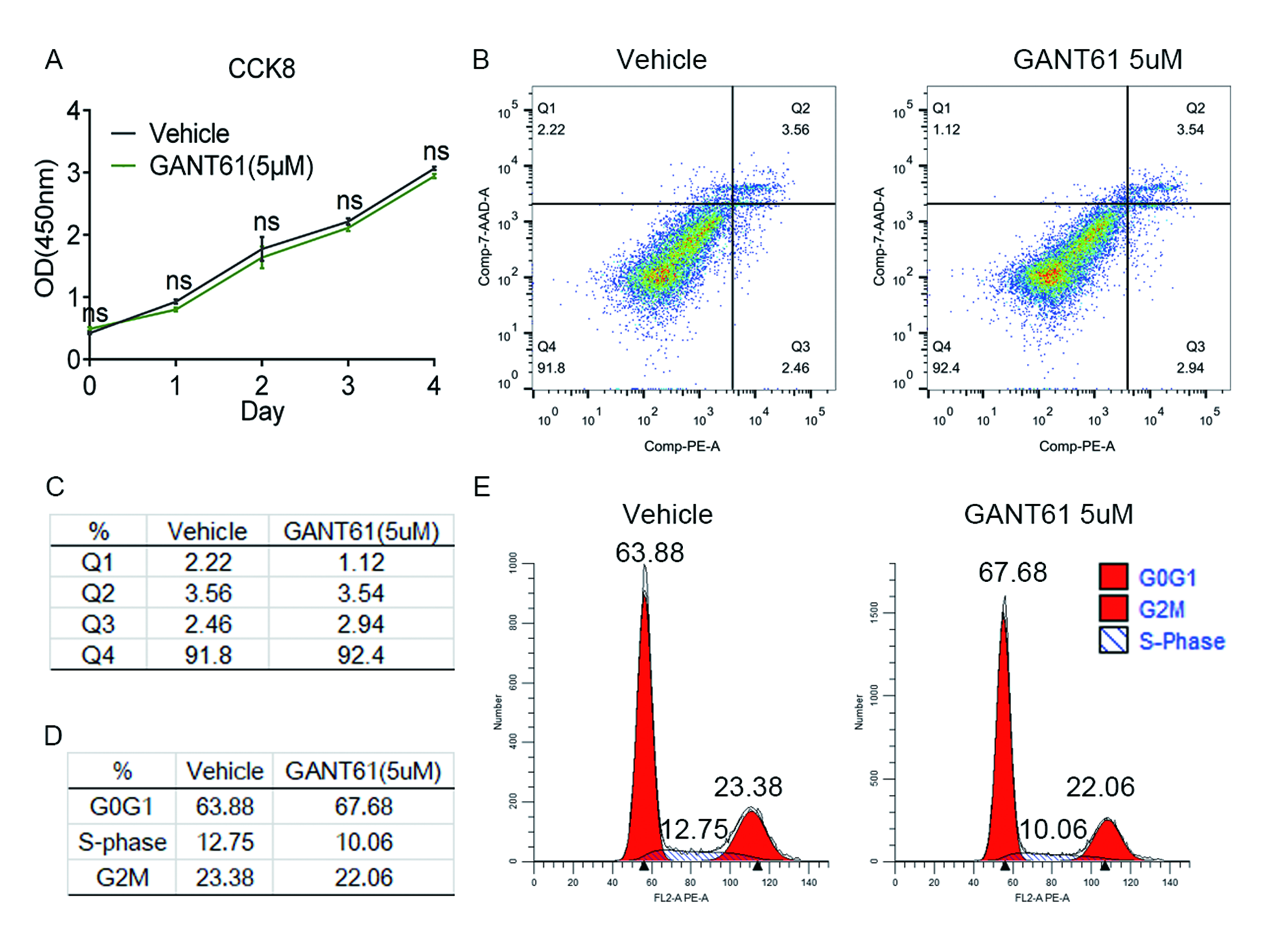

Supplement: Supplementary file 3 — Supplementary Figure 2. Treatment with 5 μM GANT61 for 24 h had no significant effect on the proliferation and apoptosis of T-HESCs. [file 41419_2020_2956_MOESM3_ESM.tif]

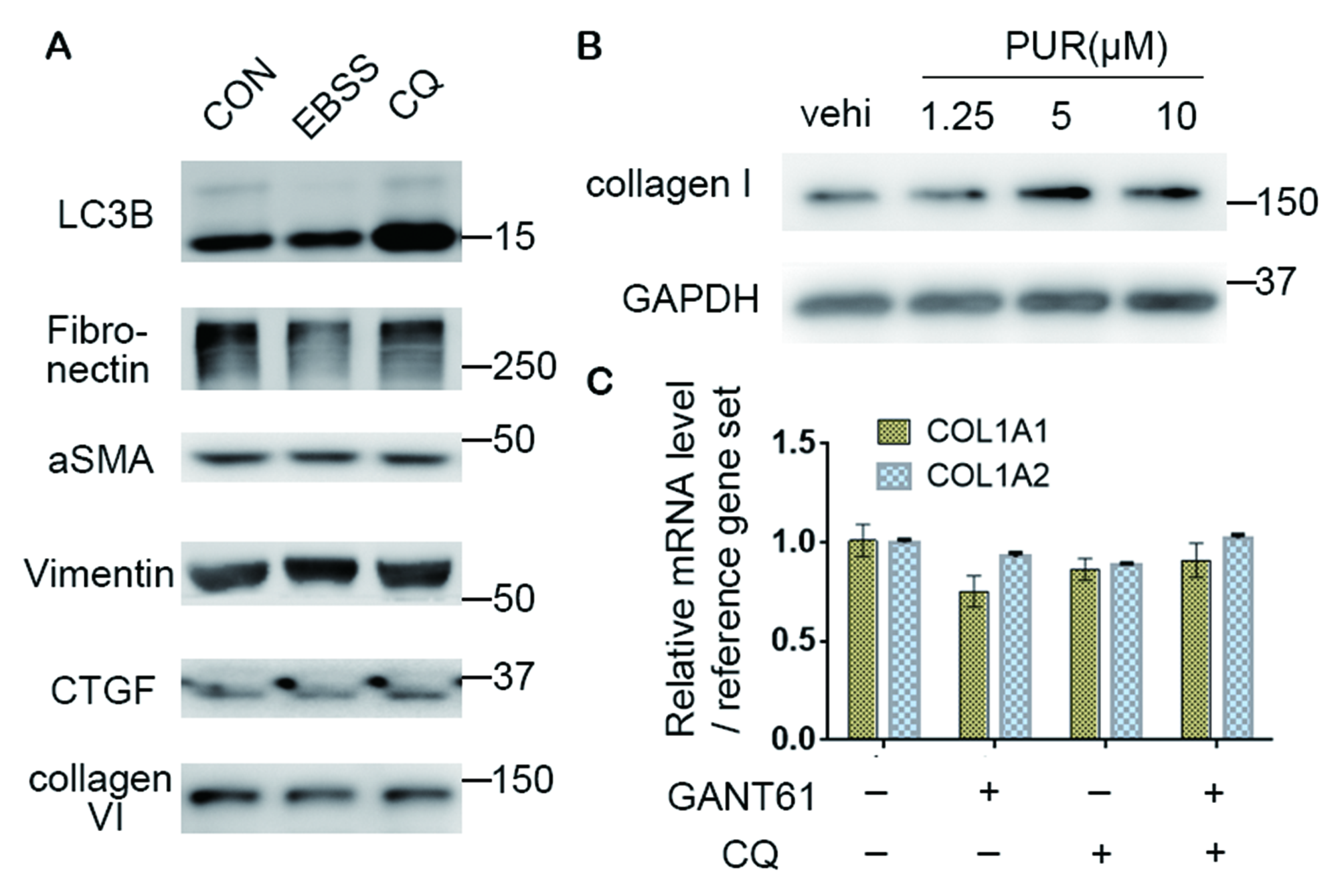

Supplement: Supplementary file 4 — Supplementary Figure 3. Important roles of Collagen I in the Hh-autophagy-fibrosis regulatory axis. [file 41419_2020_2956_MOESM4_ESM.tif]

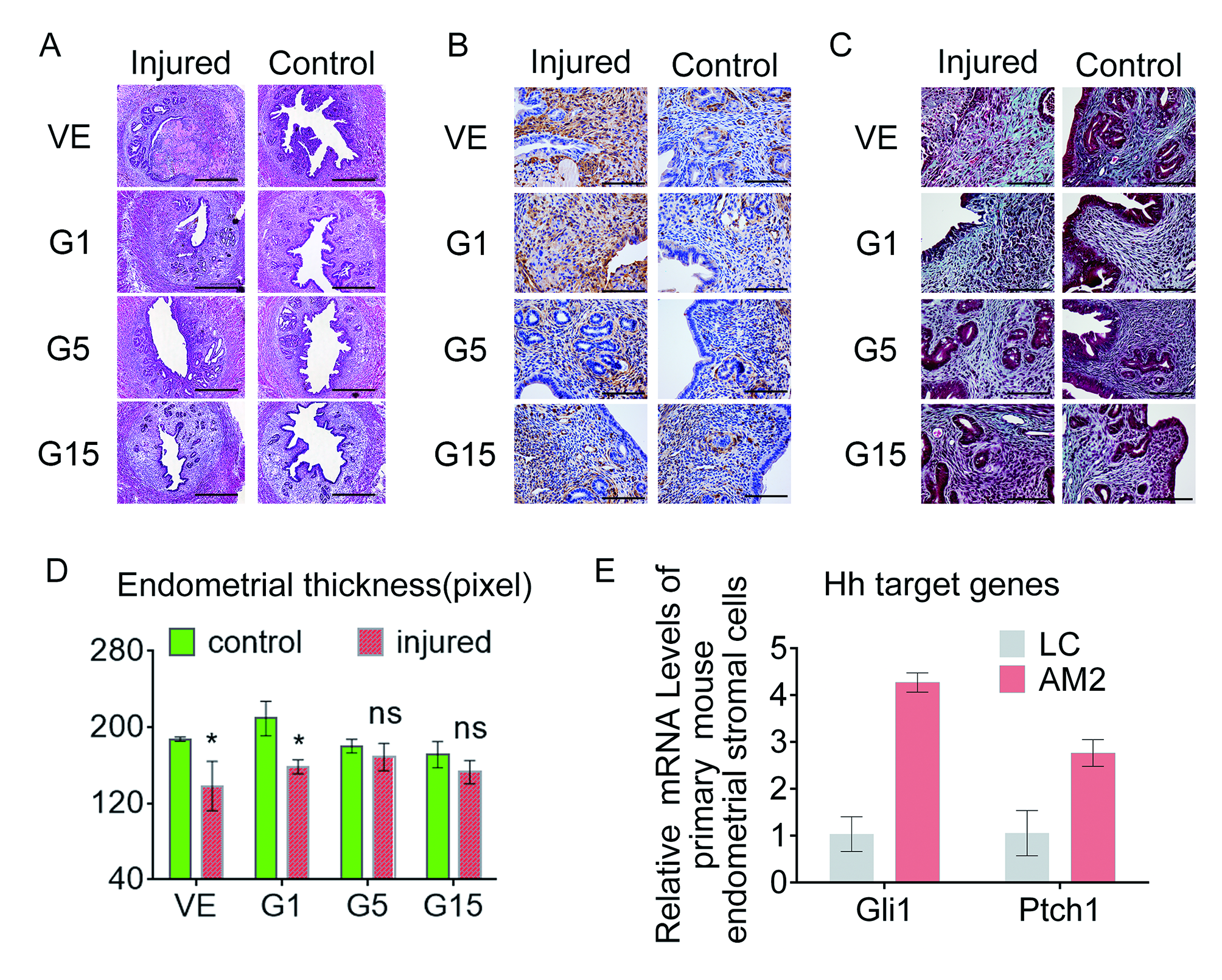

Supplement: Supplementary file 5 — Supplementary Figure 4. GANT61 reduced endometrial fibrosis in a dose-dependent manner in the murine IUA model. [file 41419_2020_2956_MOESM5_ESM.tif]
